# Ecosystem services sustainability in the Mediterranean Sea: assessment of status and trends using multiple modelling approaches

**DOI:** 10.1038/srep34162

**Published:** 2016-09-30

**Authors:** Camino Liquete, Chiara Piroddi, Diego Macías, Jean-Noël Druon, Grazia Zulian

**Affiliations:** 1 European Commission, Joint Research Centre (JRC), Via Enrico Fermi 2749, 21027 Ispra, Italy; 2 Institute of Marine Science (ICM-CSIC), Barcelona, Spain

## Abstract

Mediterranean ecosystems support important processes and functions that bring direct benefits to human society. Yet, marine ecosystem services are usually overlooked due to the challenges in identifying and quantifying them. This paper proposes the application of several biophysical and ecosystem modelling approaches to assess spatially and temporally the sustainable use and supply of selected marine ecosystem services. Such services include food provision, water purification, coastal protection, lifecycle maintenance and recreation, focusing on the Mediterranean region. Overall, our study found a higher number of decreasing than increasing trends in the natural capacity of the ecosystems to provide marine and coastal services, while in contrast the opposite was observed to be true for the realised flow of services to humans. Such a study paves the way towards an effective support for Blue Growth and the European maritime policies, although little attention is paid to the quantification of marine ecosystem services in this context. We identify a key challenge of integrating biophysical and socio-economic models as a necessary step to further this research.

Ecosystem services (ES) are the contribution of natural ecosystems to human well-being, for instance in the form of food provision, protection from flood events or inspiration for science and arts. Since ecosystems play a basic role for human societies^1^,^2^, ES have been commonly used to raise awareness of biodiversity conservation and broader ecosystem health^3^. In particular, the ES concept has been created to promote a rational and balanced measure of the use of natural resources taking into account both public and private benefits. Thus, assessing ES becomes important for fostering the sustainable management of the environment across its different functions and across multiple planning sectors.

In addition, evaluating ES spatially and temporally is essential for highlighting where the benefits for maritime economic sectors (and more generally for society) are, and how they might have changed in time. These are necessary steps needed to support and guide current regulations like the EU Maritime Spatial Planning Directive (Directive 2014/89/EU) and the Marine Strategy Framework Directive (Directive 2008/56/EC), although these regulations do not explicitly tackle ES. Only with this knowledge base can policy-makers have the necessary tools to make appropriate management decisions in order to guide proper sustainable Blue Growth in which all marine and maritime sectors can contribute to welfare, innovation and growth.

Despite the recent interest in assessing marine and coastal ES, so far most studies have described static systems, and thus static ES, with the result that currently spatial and/or temporal approaches that evaluate marine ES are still uncommon^4^,^5^ (e.g. only a few studies can be found and mostly at a local scale^6^,^7^). Data availability, knowledge gaps and uncertainty seem to be the major limiting factors.

Marine and coastal ES can be evaluated spatially and temporally through (a) the analysis or extrapolation of primary data like field sampling, surveys or high resolution remote sensing; (b) the use of seabed habitat maps and land use maps as a proxy for ES supply based on look-up tables or scoring factors; or (c) the use of selected models either ecological, socio-economic, bio-economic or specific for ES. The present study follows the third option, in particular using ecosystem models to describe the main processes/functions and quantify the delivery of marine ES. Such models have considerably evolved in the last decade driven by a worldwide movement towards the so-called ‘ecosystem-based management’ approach^8^. These new integrative marine ecosystem models are especially useful because of their ability to assess, through derived indicators for example, the linkages between climate and mean trophic levels of organisms within a system or the impacts of human activities on marine systems^9^,^10^. Even though there is a wide range of marine ecosystem models that could potentially be useful for analysing ES^11^, their application across disciplines remains scarce^5^.

This paper uses the outputs of well-developed and implemented coastal and marine biophysical models to provide model-derived indicators on specific ES of the Mediterranean Sea. The main objective was to assess ES both temporally and spatially, since several assessments have stated that the capacity of the Mediterranean Sea to deliver ES has declined over time as a result of human activities^12^,^13^. The motivation is to promote the sustainable use of marine resources and to support the implementation of marine policies with that goal. First, the paper introduces the conceptual framework and the different modelling approaches used to quantify five coastal and marine ES in the Mediterranean Sea; then, it presents the main results and indicators at sub-basin levels; and finally analyses the trends and sustainability of ES provision, presenting an argument for the usefulness of ecosystem models to provide indicators set in the ES context and how they may be applied to European policies. The five selected ES are probably the most relevant and best studied services in the marine environment^4^. They also cover the three major classes of ES, namely provisioning, regulating and cultural services.

## Approach and Models

ES can be assessed from multiple perspectives (e.g. biophysical, economical, social) using different tools and approaches (e.g. qualitative, expert-based, process-based). In this study we follow the so-called cascade framework (see Supplementary Information Section I) to identify the purpose and meaning of our analyses and, then, we apply different spatially-explicit and dynamic modelling approaches to quantify five marine and coastal ES. The intention of this paper is not to show linkages among the different selected models but instead how their outcomes could be used to assess ES. Our perspective is mostly biophysical with a link to the social dimension. Future steps in this analysis should include a suitable valuation of each service. The final goal of the current study was not to highlight the magnitude of the services delivered, but (1) to demonstrate how indicators derived from such models can be used to extract useful indicators using the ES concept, and (2) to analyse the trends and assess their capacity to maintain their benefits in the Mediterranean.

Table 1 summarizes the selected services and indicators classified under the cascade framework, i.e. indicating if the quantification refers to the natural capacity of the ecosystem to deliver services, to the actual flow of the service to humans, or to the benefit received by people. All the described indicators refer to ecosystem processes that occur across habitats and across the sediment-water, air-sea and land-sea interfaces, and are thus not limited to a specific habitat type. The indicators represent averages of three selected time periods that vary per model type (see Table 1). The modelling approaches used for the estimation of the indicators are described in the following sub-sections.

### Food provisioning: Food web model

Piroddi *et al*.^14^ constructed a food web model for the Mediterranean Sea based on the Ecopath with Ecosim and Ecospace approach (EwE, http://ecopath.org/). In particular, the static mass-balance module, Ecopath, hosts four sub-models corresponding to the main Mediterranean sub-regions (Western, Adriatic, Central and Eastern sub-basins). In total, these sub-models comprise more than 100 functional groups ranging from phytoplankton to top predators. For detailed information about the functioning of the model and its basic input parameters, please refer to the Supplementary Information Section II.1. The focus here is on the spatio-temporal analysis of the model where Ecopath is used as a baseline (representing the ecosystem in the 1950s) and Ecosim/Ecospace, the dynamic and spatial modules, are applied together to assess the evolution of the Mediterranean marine ecosystem to environmental and fishing stressors from 1950 to 2010 (this is work in progress by Piroddi *et al*.). The indicators used here are done bearing in mind that the spatially explicit analysis of Ecospace is still under development.

In this paper, we extracted indicators from EwE as proxies for fish provisioning representing the structure and exploitation status of the Mediterranean Sea ecosystem. Specifically, we extracted:

- The predicted biomass (BM; t km^−2^) of European pilchard + European anchovy (together), and European hake, the most commercially important species caught in the basin, as proxies of food provision capacity. They represent the abundance of available fish that could be used for human consumption.

- The observed catches (Cat; t km^−2^ yr^−1^) of the same species and the predicted trophic level of the catch for all retained species (TLC; scale from 1 smallest to 4 largest) as proxies of the flow of food provision. These indicators represent the actual exploitation rate of fish and its size, a characteristic of the populations’ status.

These indicators are provisional, as final temporal and spatial outputs of the model are still under development and caution should be taken when interpreting the results. The purpose of the inclusion of these indicators in this study is to demonstrate the applicability of such a model to assess ES, highlighting the indicators and the methodological approach to be used rather than focusing on the quantitative aspects of the model results.

### Water purification: Hydrodynamic & biogeochemical models

The 3-D General Estuarine Transport Model (GETM) was used to simulate the hydrodynamics in the Mediterranean Sea. GETM solves the three-dimensional hydrostatic equations of motion applying the Boussinesq approximation and the eddy viscosity assumption^15^. A detailed description of the GETM equations can be found in ref. 16 and at http://www.getm.eu. The GETM configuration for the Mediterranean Sea has a horizontal resolution of 5′ × 5′ and includes 25 vertical sigma-layers and 37 rivers discharging along the Mediterranean coast. The values for river discharges are derived from the Global River Data Center (GRDC, Germany) database while nutrient loads (nitrate and phosphate) of freshwater runoff are obtained from ref. 17. The basic hydrographic characteristics of the Mediterranean Sea such as sea surface temperature and surface currents are reasonably well simulated by this hydrodynamic model^18^,^19^.

GETM is online coupled to the MedERGOM biogeochemical model^20^,^21^ using the Framework for Aquatic Biogeochemical Models (FABM, https://sourceforge.net/projects/fabm/). MedERGOM is a modified version of the ERGOM model^22^ specifically adapted to represent the conditions of the Mediterranean ecosystem. The GETM-MedERGOM model has been used to describe present^20^, past^21^ and future^23^ biogeochemical conditions in this semi-enclosed basin. Besides the pelagic components of the marine food web (from nutrients to zooplankton, including phytoplankton and detritus) MedERGOM also simulates the detritus burial into the sediment and its re-suspension by strong currents. However, no transformation of organic matter within the sediment is currently included in this model.

For this study we selected three outputs from the GETM-MedERGOM coupled models to represent the water purification ES. This service, also known as the ‘mediation of waste, toxics and other nuisances by ecosystems’, can include processes such as filtration, sequestration, storage, accumulation, decomposition or dilution. We considered the discharge of excessive nutrient loads to be the major source of pollution occurring in the Mediterranean Sea. Thus, the selected indicators for water purification are:

- The kinetic energy of surface waters (EKE; m^2^ s^−2^) as a proxy of the capacity of water purification, since surface energy contributes to disperse pollutants.

- The nutrients uptake through primary production (PPR; mmolN m^−2^ d^−1^) as a proxy of the short-term service flow, together with the detritus burial into the sediment (Sed; mmolN m^−2^) as a proxy of the long-term or service flow. Both the uptake and the burial of organic matter are efficient nutrient removal processes[Fig f1].

### Coastal flood and erosion protection: Coastal protection model

Liquete *et al*.^24^ built a geographic model to assess the ES related to coastal protection, designing specific indicators for capacity, flow and benefit. Coastal protection was defined as the natural defence of the coastal zone against inundation and erosion from waves, storms or sea level rise, including all habitat types but excluding human-made structures which cannot be considered as ES. The study area is the coastal zone potentially affected by extreme hydrodynamic conditions (originally defined and delimited in ref. 24). It is divided into 688 coastal stretches or operational units approximately 30 km long where all the results are aggregated and normalised[Fig f2].

Compared with the previous work of^24^, this model: (1) is applied at a different spatial-temporal scale, i.e. it analyses the Euro-Mediterranean area during the years 1990, 2000 and 2010 (while the original model was static); (2) produces indicators with continuous data (while previously indicators were ranked); and (3) extends the analysis to include Croatia. The main input parameters were extracted from different models or continental databases (see Supplementary Table S3). The methodology used to process the data is consistent with Fig. 3 of^24^ and it is detailed in the Supplementary Information Section II.2. For this study, the main outputs of the model are:

- CPcap: The natural capacity that coastal ecosystems possess to protect the coast against inundation or erosion. It is based on coastal geomorphology, slope and the level of protection of both emerged and submerged habitats present. This is the selected indicator for service capacity that reflects the presence of natural features able to attenuate waves and currents.

- CPexp: The natural exposure or predicted need of protection based on the climatic and oceanographic conditions of each area. It is based on the oceanographic conditions, namely wave regime, storm surge, tide and relative sea level rise.

- CPsup: The level of supply or delivery of coastal protection that integrates natural capacity and exposure. This indicator analyses if there is enough capacity to deal with the existing oceanographic conditions, thus indicating the service flow.

- CPdem: The estimated human demand for protection in the coastal area based on the presence of residents and assets (artificial surface and cultural sites) in the coastal zone. This is the selected proxy for service benefit.

### Lifecycle maintenance: Fish habitat models

Ecological niche models are spatially-explicit methods that use the ecological requirements of a given species to predict its potential distribution in space and time. To quantify lifecycle maintenance, we used the results of two different ecological niche models centred on the reproductive aspects of fish. The first model analyses the favourable feeding conditions of the year-0 group (or 0 to 15 cm total length) European hake, *Merluccius merluccius*. The second model focuses on the preferred spawning conditions of a high market value fish, the Atlantic bluefin tuna *Thunnus thynnus*, which was recently overexploited in the Mediterranean Sea. The main steps of the modelling development (fully described in refs 25 and 26 and the Supplementary Information Section II.3) include an analysis of the main ecological traits of the species, environmental data collection and processing, and statistical analysis to identify the most favourable habitats.

The biotic (productive fronts) and abiotic conditions that define the ecological niche models were mapped daily across the Mediterranean Sea. The areas that more frequently met all the environmental requirements were identified to be the most suitable habitats for hake nurseries and tuna spawning respectively. The final suitability maps show the relative frequency of occurrence of favourable habitat (presented in days over the total of days where habitat was estimated). The two habitat models were calibrated and validated. Note that the outputs relate to potential habitat in relation to environmental conditions, and no accounting has been made for factors such as mortality by predation or fishing.

The analysis of nursery or spawning functions as ES should be differentiated both from a general assessment of ecosystem condition and from the food provisioning service^27^. The analysis of lifecycle maintenance in this study refers to species of direct use for humans, thus avoiding controversies with the general ecosystem condition. Also, both the indicators and assessment methods that characterise this ES avoid the duplication with fisheries.

The ecological niche models provide the following indicators for lifecycle maintenance:

- The frequency of occurrence of spawning habitat for bluefin tuna (BFT hab; %) and nursery habitat for hake (HK hab; %) as proxies of lifecycle maintenance capacity. The higher the frequency the more favourable habitat for reproduction for those two commercial species.

- The biomass index of hake recruits (HK BMI; kg km^−2^) as a proxy of service flow. This is an empirical index (based on observations) that indicates the actual abundance of fish surviving and potentially entering the fishery.

### Recreation: Recreation model

Paracchini *et al*.^28^ developed a model to assess nature-based outdoor recreation on European terrestrial ecosystems. This model has been applied in a multi-scale (tiered) approach^29^ and also in Barcelona^30^. The framework covers the three cascade components: (1) a capacity indicator, the Recreation Potential Indicator (RPI) which estimates the capacity of ecosystems to support nature-based recreation activities, (2) a measure of the flow of service, the Recreation Opportunity Spectrum (ROS) and (3) the demand in the form of potential trips to areas with different levels of service. The RPI included an analysis of ecosystems and features that provide opportunities for nature-based recreation activities. The computation, which has been updated for this paper, depends on four components based on the logic reported in ref. 28: (1) Suitability of land to support recreation (land use/land cover classes scored for recreation); (2) Natural features including presence and typology of natural protected areas and presence of grassland in agricultural areas; (3) Water, in the form of distance from water bodies and coast, and presence of natural riparian areas; and (4) Presence of green urban areas. The ROS integrates qualitatively the RPI with a zoning map of Europe in terms of remoteness (distance from residential areas) and proximity (distance from roads). The distribution of expected demand for local recreation estimates the share of population that can potentially access the different ROS zones, according to a spatial impedance function^31^.

In this study we have tailored the terrestrial recreation model, run it for the years 1990, 2000 and 2010 (approximately, because the last land cover data set is for 2006), and developed a new module to focus on coastal recreation. The new marine component, which takes part of the RPI, includes coastal geomorphology, the ecological status of transitional and coastal waters, and water transparency (see data sources and processing in the Supplementary Information Section II.4). These are considered three of the key characteristics of coastal ecosystems that attract people to use recreational areas. The new RPI gives 50% weighting to the terrestrial RPI and 50% to the new marine component. Following this, the EU was divided into zones depending on remoteness from roads and proximity to urban areas. This EU zoning is cross-tabulated with RPI to estimate the ROS (see Supplementary Table S5). We do not estimate the recreation demand in this work. The reporting units in this analysis are the coastal Local Administrative Units (LAUs) level 2 that consist of municipalities or equivalent units in the 28 EU Member States which touch the coastline. The spatial analysis extracts average values of all the variables in each of the LAUs inland (for the terrestrial component and coastal geomorphology) and up to 10 km offshore (for the marine parameters). The final indicators for recreation are aggregated by coastal LAUs and represent:

- The capacity for nature-based recreation in the coastal zone, covered by the combined RPI for terrestrial and marine habitats.

- The potential flow of recreation, estimated by the ROS indicator as a combination of natural capacity and accessibility.

## Results

**Figure 1 f1:**
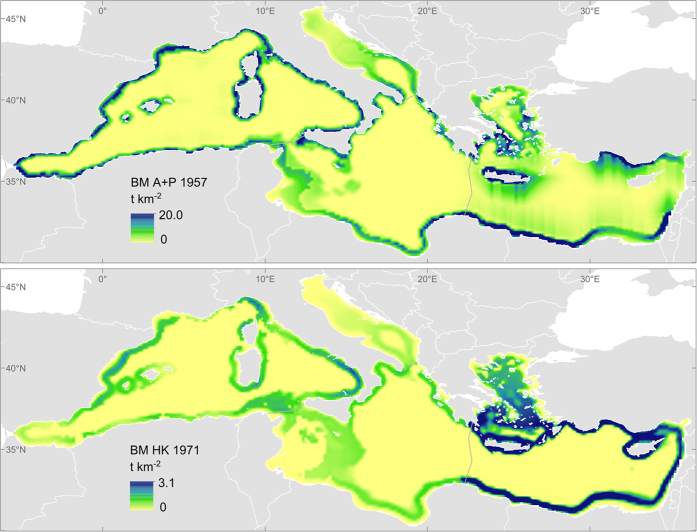
Preliminary results for one single year from the Ecospace (spatial) module as an example of the distribution of indicators. BM A+P: Biomass of European anchovy + European pilchard, indicator of the capacity of food provisioning. BM HK: Biomass of European hake, indicator of the capacity of food provisioning. The temporal trends for all the indicators (biomass, catch and trophic level of catch) are estimated by the EwE model at sub-basin level. Maps generated with ArcGIS 10.2.2 for desktop (http://www.esri.com/software/arcgis).

**Figure 2 f2:**
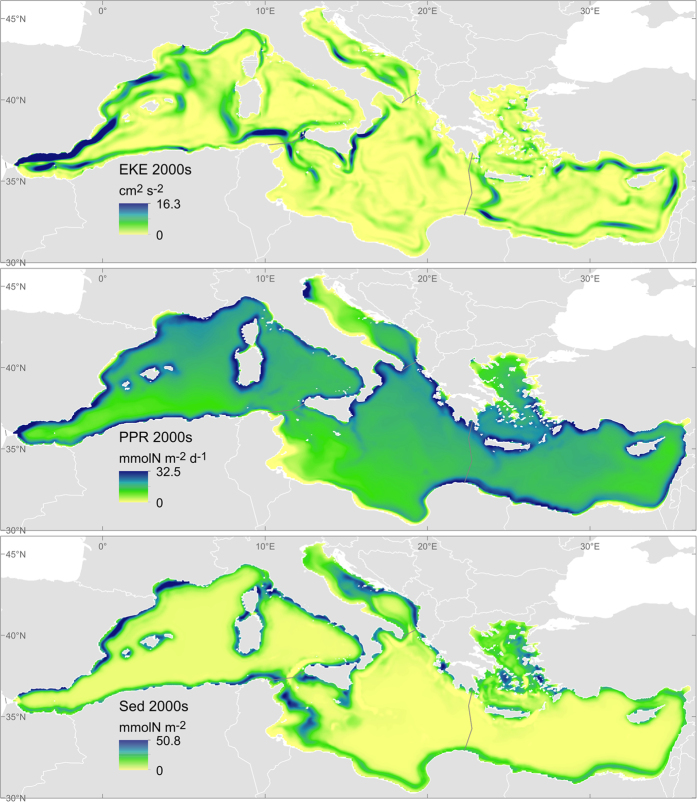
Distribution of the water purification indicators for the last available period, 2000–2010. EKE: Kinetic energy of surface currents, indicator of the capacity of water purification. PPR: Primary production, indicator of the short-term flow of water purification. Sed: Burial of organic matter into the sediments, indicator of the long-term flow of water purification. Maps generated with ArcGIS 10.2.2 for desktop (http://www.esri.com/software/arcgis).

**Figure 3:**
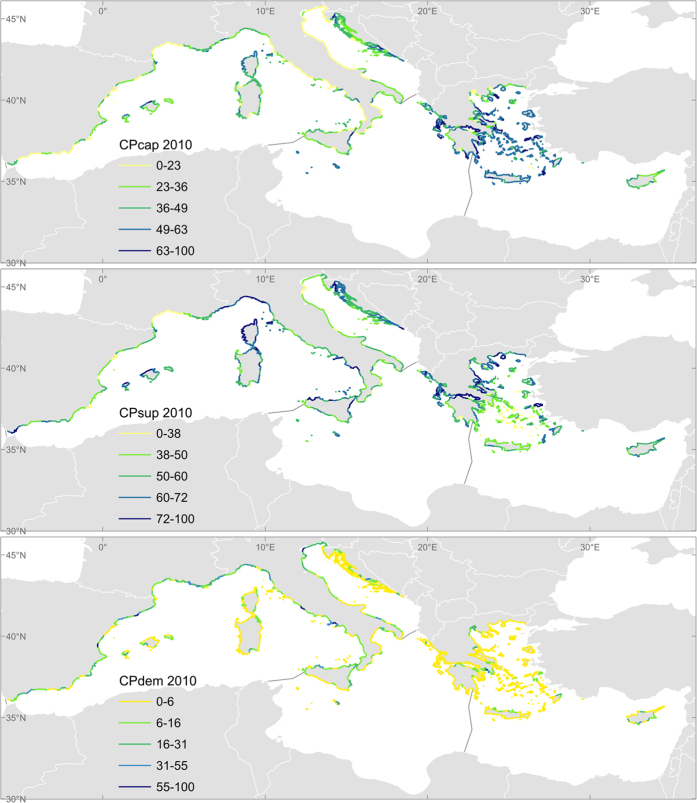
Distribution of the coastal protection indicators for the last available period, 2010. CPcap: Coastal protection capacity, indicator of the natural capacity to provide protection. CPsup: Coastal protection supply, indicator of the flow of coastal protection. CPdem: Coastal protection demand, indicator of the benefit from coastal protection. Maps generated with ArcGIS 10.2.2 for desktop (http://www.esri.com/software/arcgis).

**Figure 4:**
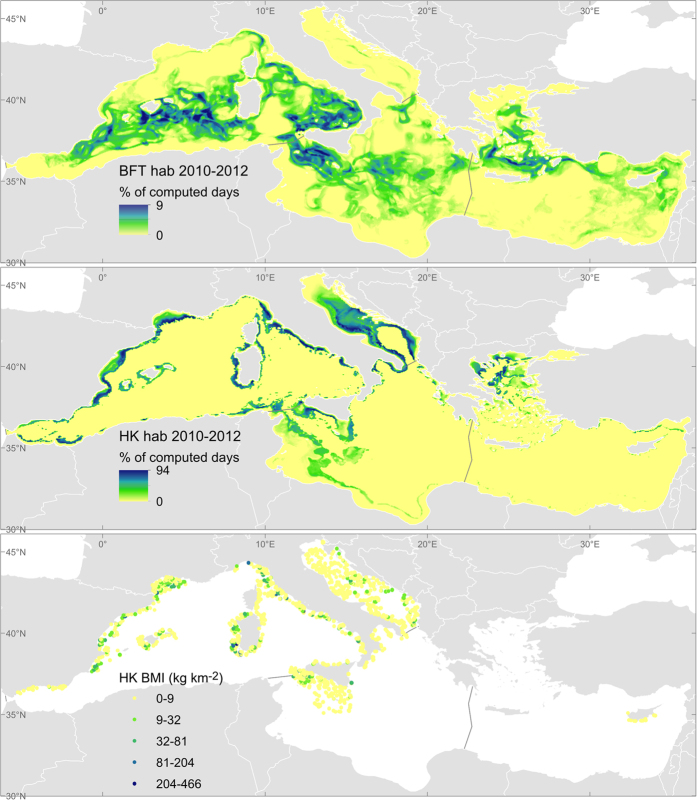
Distribution of the lifecycle maintenance indicators for the last available period, 2010–2012. BFT hab: Favourable spawning habitat for bluefin tuna, indicator of the capacity of lifecycle maintenance. HK hab: Favourable nursery habitat for European hake, indicator of the capacity of lifecycle maintenance. HK BMI: Hake biomass index, indicator of the flow of lifecycle maintenance. Maps generated with ArcGIS 10.2.2 for desktop (http://www.esri.com/software/arcgis).

**Figure 5:**
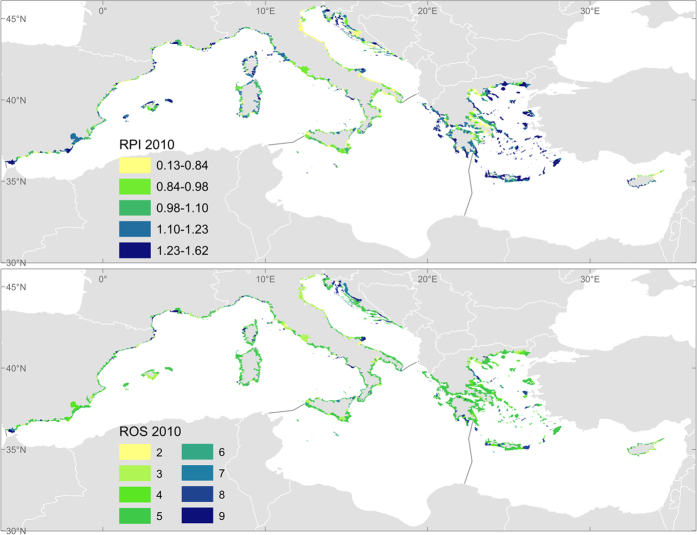
Distribution of the coastal recreation indicators for the last available period, 2010. RPI: Recreation potential index, indicator of the capacity for nature-based recreation. ROS: Recreation opportunity spectrum, indicator of the flow of recreation (2 is the minimum and 9 the maximum flow, see legend in Supplementary Table S5). Maps generated with ArcGIS 10.2.2 for desktop (http://www.esri.com/software/arcgis).

A summary of the results of the selected indicators per ES is provided in Table 2. Each sub-section discusses first the indicators of capacity, followed by those of service flow and, if available, benefit. For each indicator we elaborate on the time trends and on the spatial distribution. The spatial dimension discussed here focuses on the four Mediterranean sub-basins although higher resolution analyses could be performed with the selected models. Figures 1, 2, 3, 4 and 5 show examples that illustrate the results, in particular the most recent quantification of each indicator, while the remaining analysed periods and the spatial changes are mapped in the Supplementary Information Section III. The only exception is the spatial analysis of food provisioning which is still an ongoing work and results are not definitive yet.

### Food provisioning: wild fish

The capacity of the Mediterranean Sea to provide wild fish for consumption can be explored through the estimation of total biomass of commercial species. The biomass of European pilchard + anchovy (BM P+A) and of European hake (BM HK), three of the most important commercial species in the Mediterranean Sea, seems to follow similar temporal trends in each of the sub-basins (Table 2). In the Western Mediterranean there is a continuous decrease in biomass from the 1960s to the 2000s; in the Adriatic there is a general decrease that was interrupted in the 1980s; while in the Central and Eastern Mediterranean there is a general increase at least from the 1960s to the 1980s that has since become stable or changed trend. The three species distribute mainly along the continental shelf of the Mediterranean Sea. Hake seems to be more abundant in the Eastern basin, and pilchard and anchovy in the Western and Eastern Mediterranean Sea. These results come from the four Ecopath with Ecosim Mediterranean sub-models, while the Ecospace spatial model results are still under development and only one-year samples are shown in Fig. 1.

The flow of fish provisioning service is analysed through the catches (Cat) of these important fish species. In this case the trends are similar across sub-basins (Table 2). In all cases there is a peak of fish catch in the 1980s that is generally double that of the 1960s. This trend is reversed when compared to the trophic level of catch (TLC), an indicator of exploitation levels, although this indicator can be labelled as stable due to the relatively small variations observed, which were only found to be significant in the Adriatic Sea. From the 1980s, the catch decreases in all basins except the Eastern Mediterranean. The total catch is particularly high in the Western Mediterranean and, for pilchard and anchovy, in the Eastern basin. This means that capacity (biomass) and flow (catch) are not necessarily in synchronicity.

### Water purification: nutrients retention

The capacity of surface waters to clean or disperse pollutants can be approximated with the intensity of surface circulation (kinetic energy). This energy is especially high in the Western Mediterranean and tends to be concentrated in mesoscale oceanographic features (see Fig. 2). In this sub-basin the kinetic energy decreased from the 1960s to the 2000s (especially from the 1960s to the 1980s) while in the rest of the Mediterranean it shows a more or less continuous rise (Table 2).

The actual cleaning of nutrients is quite different both spatially and temporally if we consider short-term processes (reflected by PPR) or long-term processes (reflected by Sed). PPR concentrates in coastal waters, is more intense in the Western basin, undergoes a sudden increase from the 1960s to the 1980s and then stabilizes afterwards, although continues to increase slightly, particularly in the Eastern Mediterranean. Such observations could be linked to the input of excess nutrients from land. In addition, the total fish catch per unit effort in the Mediterranean shows a trend similar to PPR^20^. The long-term retention of organic matter in the sediments is relatively stable and tends to decrease (at least since the 1980s) in all the sub-basins except for the Adriatic, where it shows a sharp increase notably until the 1980s. The shallow and enclosed nature of this sub-basin together with the increase in PPR (mostly linked to an increase of productivity/pollution coming from the Po river plume) could explain of such trend.

### Coastal flood and erosion protection

The capacity of coastal habitats to protect people and assets from inundation and erosion, reflected in the CPcap indicator, decreased between 1990 and 2010 around the entire Mediterranean Sea (although with very low intensity in the Adriatic). This situation does not seem to have improved in recent years (Table 2 and Fig. 3). Land use and shoreline change are the leading variables behind this trend. The Eastern and Central basins are more protected than the Western and, especially, the Adriatic shores.

The actual supply or flow of coastal protection (CPsup) has maximum values around the Central and Western Basins where CPsup suffered the sharpest decrease from 1990. The drop of CPsup was not found to be as intense as in the other two sub-basins, although in the Eastern Mediterranean the situation does show a declining trend in recent years.

The demand of coastal protection (CPdem), indicative of the benefit of the service, grows all across the Mediterranean Sea, notably in the Western part that already shows the highest demand. These growing trends combined with the decline of CPcap and CPsup should raise concern for coastal communities.

### Lifecycle maintenance: spawning and nursery habitats

The two types of indicators used to assess the capacity of lifecycle maintenance (the distribution of spawning habitats for bluefin tuna or ‘BFT hab’ and nursery habitats for the European hake or ‘HK hab’, Table 2 and Fig. 4) reflect the complexity of this service as both proxies behave differently over the analysed time period (2003–2012). In the Western Mediterranean, the capacity to maintain the lifecycle in that period increased for tuna while it declined slightly for hake. In general, the occurrence of the two types of habitats appears be stable in the Adriatic (where the spawning habitat for tuna is marginal) and shows a decline in the Central and Eastern basins. The complexity of this service is due to the different life stages analysed and the distribution of core habitats (pelagic vs demersal) which are affected by different environmental covariates or dynamics^25^,^26^. A longer time series for such indicators would be advantageous to determine a consistent trend. The core reproductive habitat is nevertheless well assessed spatially and seasonally and can be used to monitor this ES. The southern part of the Central and Western Mediterranean are the most extended and recognized spawning grounds for bluefin tuna while the Adriatic Sea and the shelf-break area are well known as the preferred nurseries for hake.

The flow of lifecycle maintenance is represented in this study by the distribution of hake recruits’ biomass (HK BMI), an index elaborated from observed data across the Mediterranean (MEDITS) from 2003 to 2011. In general, this biomass index is higher in the Western Mediterranean and tends to decrease in all sub-basins, especially from 2006–2008 to 2009–2011 (Table 2 and Fig. 4). The only exception is the Eastern Mediterranean, where the data series are too poor to extract a reliable trend.

### Recreation: coastal opportunities

The capacity for nature-based recreation shows a slightly decreasing trend in all Mediterranean sub-basins, although this trend can be considered as stable since the change represents less than 1% of the original values (Table 2). In any case, the slight decay tends to be concentrated in the final years of the analysis and is mostly due to the marine component (which is linked to coastal geomorphology and water transparency changes). There are also decreasing though trivial trends in the terrestrial component of the indicator driven by land cover changes. In none of the cases (per component or per sub-basin) are increasing trends observed.

The flow of recreational use for coastal communities seems stable across the Mediterranean region (Table 2 and Fig. 5). The fixed category of the Recreation Opportunity Spectrum indicator (ROS class = 5) corresponds to medium capacity and proximal opportunities (see Supplementary Information Section I). The categorical nature of the indicator and the large scale of the analysis (per sub-basin) hide the small-scale variability, highlighting that the trend of this ES should be analysed with higher resolution or smaller scale studies. With this in mind, an update of the original recreation model integrating the influence of some coastal parameters in the terrestrial output (at 100 m resolution) is under preparation. It must be noted that tourism (i.e. long distance and/or time travel for leisure usually for stays of more than one day) as well as recreational activities based on human infrastructures are not covered by these recreation indicators.

## Discussion

### Modelling marine ecosystem services

Many ecosystem models explore the biodiversity and ecosystem functioning of the ocean^11^. However, this rapidly evolving modelling science is not yet commonly applied to assess the delivery or benefit of ES. The spatial and temporal scales over which marine ecosystem models are built qualify them for representing an efficient tool to study long-term sustainability and support knowledge-based management through the analysis of options or scenarios.

The results of this paper show that spatial modelling approaches can be integrated in ES’ frameworks to provide critical assessments. This can also be very useful for the development of marine ecosystem accounts, the attempt to integrate environmental information into standard measures of economic activity or national economic accounts (e.g. http://unstats.un.org/unsd/envaccounting/seea.asp). A few similar approaches have been applied for instance in Belize^32^, the USA^33^,^34^,^35^,^36^,^37^, Canada^38^, South Corea^39^, the Mediterranean Sea^40^ and also globally^41^. These authors relied on EwE models^34^,^37^,^41^, InVEST models^32^,^38^,^39^, specific ecological-economic models^33^, biogeochemical models^40^ or specific functional models^35^,^36^ to quantify a variety of ES. Large scale (i.e. more than supra-national) assessments are still very rare. Our approach makes a broader use of different models compared to these examples, and clearly classifies each of the ES’ indicators and measures obtained, although a direct link with valuation studies (either monetary, non-monetary or based on value pluralism) is absent. This is in part due to the large scale and spatial nature of the analysis that challenges the application of socio-economic assessments.

Together with these modelling approaches, there is a growing interest on the analysis of the influence that water or habitat quality may have on the delivery of ES, either on the perception of individuals or on the broader recreational use of marine and coastal areas^39^,^42^,^43^,^44^. The effect of drivers and pressures on ES is indeed one of the most relevant topics for management support. In those studies, the focus is on the use of economic techniques with limited use of modelling approaches.

### The sustainability of ecosystem services provided by the Mediterranean Sea

The flow of fish provisioning, or what the fisheries extract from the sea, is increasing across the entire Mediterranean Sea (Table 3). This is supporting important economic benefits, rates of employment and a healthy protein diet but, as with any other provisioning service, it also exerts pressure on the marine environment. The natural capacity to sustain this flow of fisheries is decreasing at least in the Western and Adriatic seas (Table 3), which points to important sustainability issues. The results shown in this paper provide a general overview of the Mediterranean ES, however more detailed analysis at management scale and development of future scenarios using such modelling approaches are needed for decision making. Tsikliras *et al*.^45^ used a catch-based method and similar indicators to ours (e.g. landings and trophic level of catch) to conclude that all Mediterranean fisheries resources are at risk from overexploitation, with the Eastern basin being the worst. This is in agreement with our results since our fishing capacity trends also show an important decline on the timespan analysed by^45^, from the 1970s to present (see Table 2). Similarly^34^,^37^, selected the EwE biomass as an indicator of fishing capacity while^41^ focused on the model fit for catches. Modelled catches are not shown in this paper but are part of the outputs of the Mediterranean EwE model and will allow for making predictions of fisheries in forthcoming studies. Also, the inclusion of key habitats (like seagrass beds) or species of interest (like the most commercially targeted) as functional groups in the food web model (see Supplementary Table S2) allows for capturing the different responses that individual species or groups have to environmental changes, pressures, trade-offs or level of ES delivery^34^,^37^.

The actual removal of nutrients or flow of the water purification service is rising across the Mediterranean or has an undefined trend (in the Western sub-basin, Table 3). An important point to consider is that the indicators behind this service include the input from land sources, meaning that the natural retention of nutrients can be proportional to the input of human pollution and, thus, they may not reflect the sustainable flow of the service. The notion and importance of sustainability indicators for water-related ES, especially when high flow or benefit rates can lead to overexploitation, is discussed in ref. 46. The hydrodynamic conditions appear to enhance the natural capacity to disperse pollutants in all sub-basins except in the Western Mediterranean (Table 3). Although the link between water quality issues and ES is a hot research topic (see previous section), there are very few studies modelling water purification as a service of marine environments. Examples of such include the analysis of filtration capacity by oyster populations at the estuary level^35^ and the economic valuation of water purification at a tropical island^47^, both of which are local coastal case studies. As abovementioned, our indicators of water purification still do not capture the sustainability thresholds or the socio-economic value, but do provide a valuable starting point to assess accurately, both temporally and at large spatial scale the provision of this service.

Both the capacity and the actual supply of coastal protection are decreasing all along the Mediterranean shores, except in the Adriatic Sea where it is estimated to be stable (Table 3). At the same time, the human demand for protection is growing in the entire region which may lead to unsustainable or more risky situations. Such a situation could be mitigated with hard defence structures, but these structures usually neglect long-term sustainability^48^ and disturb sediment dynamics and ecological processes^49^. Two InVEST models (‘Coastal Vulnerability’ and ‘Erosion Protection’) provide a comparable approach to the model used in this study, although they focus on the presence of a few habitats^32^,^39^,^50^. Arkema *et al*.^50^ conclude that the loss of certain coastal ecosystems would lead to a great damage to people and properties and to massive investments. Numerous indicators have been proposed to set a monetary value to coastal protection (see compilation in ref. 4) although they are not usually connected to the natural capacity or supply of the service. An exception is Vassallo *et al*.^51^ who provide a specific quantification and valuation of the sediment retained by *Posidonia oceanica* in the Mediterranean.

The natural capacity to maintain the reproduction of tuna and hake show decreasing trends in the Eastern and Central Mediterranean Sea and it is relatively stable in the Adriatic and in the Western basin (Table 3). It is however difficult to draw clear conclusions from our relatively short time series. Bearing this in mind, the actual flow of lifecycle maintenance reflected by hake survival rates and renewed biomass showed a substantial decrease across the Mediterranean Sea (with a mean decrease of 44% over the sub-basins and an unknown trend in the Eastern basin), pointing to substantial sustainability issues. Previously the assessment of the spawning/nursery functions as an ES has been focused on coastal habitats and local case studies (see review in ref. 27). For example^36^, assessed the role of *Crassostrea virginica* reefs in the USA modelling the recruitment, growth and mortality of enhanced species. In the Mediterranean region, seagrass meadows are of special interest for lifecycle maintenance and also for estimating the proportion of fisheries benefits (either commercial or recreational) that is linked to those meadows^51^,^52^, so further effort should be dedicated to consider this coastal component as part of this service. Nonetheless the large continental shelves and the open ocean are key areas for fisheries and fish reproduction and, apart from our modelling approach, we are not aware of other methods that target the pelagic spawning or demersal nurseries habitats at the scale of the Mediterranean Sea which could be used as time series to derive trends for this indicator.

Due to the minor changes observed in the indicator of nature-based capacity for coastal recreation, the trend in the entire Mediterranean basin is considered stable (Table 3). However, analyses of individual variables and components of this indicator show slight deteriorating tendencies for recreation potential, like in the case of land cover/land use, shoreline morphology or water transparency changes. The flow of local recreation around the Mediterranean Sea is also considered constant with the metrics and scale proposed in this study, although other trends could be extracted at higher resolution. Even if the benefit of this service has not been analysed, the general increase of coastal population observed in the quantification of demand for coastal protection could imply a similar trend for recreation demand. Also, tourism is not covered by our model, but the opportunities that the natural Mediterranean environment offers for relaxation and amusement can be similar for tourists and local residents. Hence, a large part of the demand for this service may come from tourists. The number of tourists in Southern/Mediterranean European countries continue to grow during recent years, accounting for more than 200 million people or one fifth of the global market in 2014^53^. This increasing trend is forecasted to slow down and distribute seasonally in the coming decades partially due to climate change^53^,^54^. In general, the majority of publications that assess coastal or marine recreation focus on the human benefit^4^ proposing economic valuations of specific habitats^44^,^55^,^56^. In contrast, our modelling approach quantifies the capacity and flow of this service, and covers all habitat types. Similar to our approach, the InVEST Recreation model offers the opportunity to make spatially-explicit assessments of capacity and flow of service, to understand which elements of nature attract people, and to develop scenarios of ecosystem change and visitation rates^32^,^39^,^57^.

Based on the results of^40^ on the climate regulation service, the Mediterranean Sea acts as a sink of CO_2_ contributing globally more than its share of coverage, with an estimated overall flux of CO_2_ from the atmosphere to the sea of 17.8 million t/yr under current conditions. This flux shows a west-east gradient distribution with higher sink fluxes in the Western Mediterranean and lower (and even negative) rates in the Eastern sub-basin. The contribution of physical (abiotic) processes to carbon sequestration is found to be either positive or negative, while the contribution of biological processes (biological pump) is always positive and highly significant. The analysis is based on a transport-biogeochemical model developed and validated for describing plankton productivity and the carbon biogeochemical cycle in the Mediterranean. This biophysical approach is combined with various estimates of the social cost of carbon emissions that provide a carbon sequestration value of 127–1722 million €/yr for the entire Mediterranean Sea. Since multiannual trends were not available from^40^, such service was not considered in our analysis. Yet, such work provides a good example of how modelling approaches or results can be coupled with economic techniques to support the development of new ecosystem accounting systems.

Integrating all the ecosystem services analysed in this paper, the Western Mediterranean Sea is likely experiencing the most unsustainable situation due to high demand of services and is consequently under high exploitation rates and pressures. For this reason, in many cases the flow of services peaks in this sub-basin. The Eastern Mediterranean Sea is in many aspects the second largest provider of services but it is also the sub-basin showing the most severe deterioration in recent times. The Adriatic Sea has a critical importance for selected services such as lifecycle maintenance and long-term retention of nutrients in relation to its particular oceanographic characteristics (notably large continental shelf and high current velocities).

### Policy implications

ES have long been seen as a way to introduce economic aspects in conservation policies and environmental aspects in sectorial policies. They can actually play both roles since they are able to integrate environmental and socio-economic perspectives to sustainably manage natural resources. This idea is fostered by the implementation of the cross-sectoral EU Biodiversity Strategy to 2020 (COM(2011) 244 final). The application of ES in concrete coastal and marine management decisions is however still very rare probably due to the lack of common understanding of ES concepts and the lack of established methodologies to quantify them. This study demonstrates that the current scientific knowledge (specifically ecosystem and biophysical modelling) is able to perform a first evaluation of marine ES. We agree with^58^ that the present research priorities in the field of marine ES are more on the interplay between ecosystems and people (i.e. the integration of economics, natural and social sciences as well as the link between ES and well-being) than on instrumental questions.

In Europe, the environmental pillar of the maritime policy is the Marine Strategy Framework Directive (MSFD, Directive 2008/56/EC) that aims to achieve good environmental status in marine waters following an ecosystem-based approach. This Directive does not refer explicitly to ES but a number of the indicators that can be used to quantify marine ES (such as the ones shown in this paper) overlap with the MSFD attributes and/or indicators. The ecosystem-based approach of the MSFD would benefit from the inclusion of marine ES. We contribute here providing new data (indicators and results), possible assessment systems (models and frameworks) and interpretation (temporal and spatial trends) to assess some of these ES. For example, all the indicators of the ES water purification are relevant to analyse the fifth descriptor of the MSFD that deals with human-induced eutrophication. Moreover, most of the environmental data needs to analyse marine and coastal ES are already covered through the established MSFD monitoring networks. Further efforts should be devoted to the integration of socio-economic analyses (which is also missing in some of the assessments of this article) taking into account the limitations and challenges of the valuation of marine ES^59^. Thus, we advocate for the integration and clear identification of ES assessments in the next revision of the MSFD implementation, as already noted by other authors^11^,^60^,^61^.

The recent Maritime Spatial Planning Directive (MSPD, Directive 2014/89/EU) only mentions ES once and ES are not integrated into the process despite the fact that they could be a powerful tool to support a sustainable use of marine resources by facilitating the analysis of conflicts and trade-offs coming from multiple uses and impacts^38^,^62^,^63^. Even though three of the six themes of the Blue Growth (central focus of the MSPD) are closely related to ES (fishing & aquaculture, environmental protection and tourism) in our view, the Directive considers ES as part of biodiversity or environmental conservation, which is in turn limited to the designation of marine protected areas. Environmental conservation and ES should not be limited to the delimitation of “no-take zones” but to the sustainable and balanced distribution of resources for humans and nature, which is in line with the motto of the EU maritime policy “healthy oceans, productive ecosystems”. To accomplish this task, a holistic and spatially-explicit analysis of the natural capacity to deliver ES, together with the actual flow of these services to society and the benefits extracted from them should be performed, with a special focus on long-term trends and sustainability. By taking into account the entire range of uses of the sea, the beneficiaries are not only single economic sectors but also the whole human society. Again, the results and the approach shown in this paper could support or inspire MSP in the Mediterranean, highlighting the broad multi-functionality and range of benefits provided by the marine environment. A simple example is the distribution and analysis of impacts in the nursery area of hake (assessment of lifecycle maintenance) compared with the presence of adult biomass and the fishing exploitation of this species (assessment of food provisioning).

## Conclusions

The sustainability of marine and coastal ecosystem services (ES) provided by the Mediterranean Sea can be analysed through indicators that quantify capacity, flow and, if available, demand of service through time and space. Temporal analysis presented here show food provisioning and coastal protection to be the most unsustainable ES especially in the Western Mediterranean sub-basin, with the Eastern sub-basin showing signs of deterioration only in recent years. Water purification at sub-basin level can be maintained through natural processes, but it is highly dependent on the excess nutrient and pollutant inputs from land that could affect specific coastal environments (e.g. eutrophication in the Adriatic Sea). Lifecycle maintenance is relatively variable in time and space depending on the particular species under study, although it generally shows a decreasing or stable trend. Recreation can be considered stable at sub-basins level, but the natural potential to support it is slightly deteriorating and we assume that the demand will be growing. Overall, this study found more decreasing than increasing trends in the natural capacity to provide marine and coastal ES and more growing than falling trends in the realised flow of services to humans, likely driven by a higher demand. The observed decrease in ES supply and the uncoupled trend of ES flow can eventually affect the well-being of local residents and other beneficiaries.

We can also extract some recommendations based on the applicability of model-derived indicators shown in this study:

- Our results have demonstrated that ES could be assessed both spatially and temporally using different modelling approaches, helping to fill the gaps that still exist in the scientific literature and providing comprehensive information for marine spatial planning.

- Some ES are better assessed (e.g. fish provisioning) than others (e.g. carbon sequestration) and more effort should be dedicated to assess them. In this context, it is essential to analyse and model the effect of human activities and pressures on ES (e.g. land use and seabed habitat changes affecting recreation or coastal protection). Also, the integration of biophysical with socio-economic models needs to be strengthened. A particular challenge we found here is the limited availability of socio-economic spatial data.

- The usage of the framework presented here facilitates the mainstreaming of ES in current policies and management decisions. The mapping and assessment of ES is required by the EU Biodiversity Strategy, but so far it is not included in the European maritime policy. In order to make such studies applicable and implemented in current regulations, uncertainties of model outputs should be evaluated. In addition, the time scale (e.g. in the case of water purification) and spatial scale (e.g. in the case of recreation) of the analysis determines the results and the management level at which these studies can be applied.

- The spatial-temporal analysis of sustainability from some areas (e.g. Western Mediterranean) could be useful to develop management strategies in other less exploited zones (e.g. Eastern Mediterranean). Good strategies should be reflected by an improvement of ES capacity, and eventually flow and benefit. Points of special concern can be evidenced by decreasing capacity and increasing flow or demand, as in the case of Mediterranean coastal protection.

- Results presented here focused mainly on the temporal and spatial hindcasting analysis of selected ES of the Mediterranean Sea. A necessary future step in support of EU regulations would be the evaluation of different management strategies (e.g. decrease of fishing effort or reduction of nutrient loads) through forecasting analysis.

### Disclaimer

The scientific output and views expressed in this document are purely those of the authors and do not imply a policy position of the European Commission.

## Additional Information

**How to cite this article**: Liquete, C. *et al*. Ecosystem services sustainability in the Mediterranean Sea: assessment of status and trends using multiple modelling approaches. *Sci. Rep.* **6**, 34162; doi: 10.1038/srep34162 (2016).

## Supplementary Material

Supplementary Information

## Figures and Tables

**Table 1:** List of services, models and indicators applied in this research.

Ecosystem service	Model applied	Time span (t1, t2, t3)	Indicator of capacity	Indicator of service flow	Indicator of benefit
Food provisioning	Ecopath with Ecosim	1960s,1980s,2000s	Biomass of commercial species	Total catch. Trophic level of catch	
Water purification	GETM and MedERGOM	1960s,1980s,2000s	Kinetic energy	Primary production. Nutrients retention in sediments	
Coastal protection	Coastal protection (CP) model	1990,2000,2010	CP capacity	CP supply	CP demand
Lifecycle maintenance	Various habitat models	2003–05,2006–09,2010–2012	Potential spawning habitat of bluefin tuna. Potential nursery habitat of hake	Biomass of (survived) hake recruits	
Recreation	ESTIMAP-Recreation	1990,2000,2010	Recreation Potential Indicator	Recreation Opportunity Spectrum	

**Table 2:**
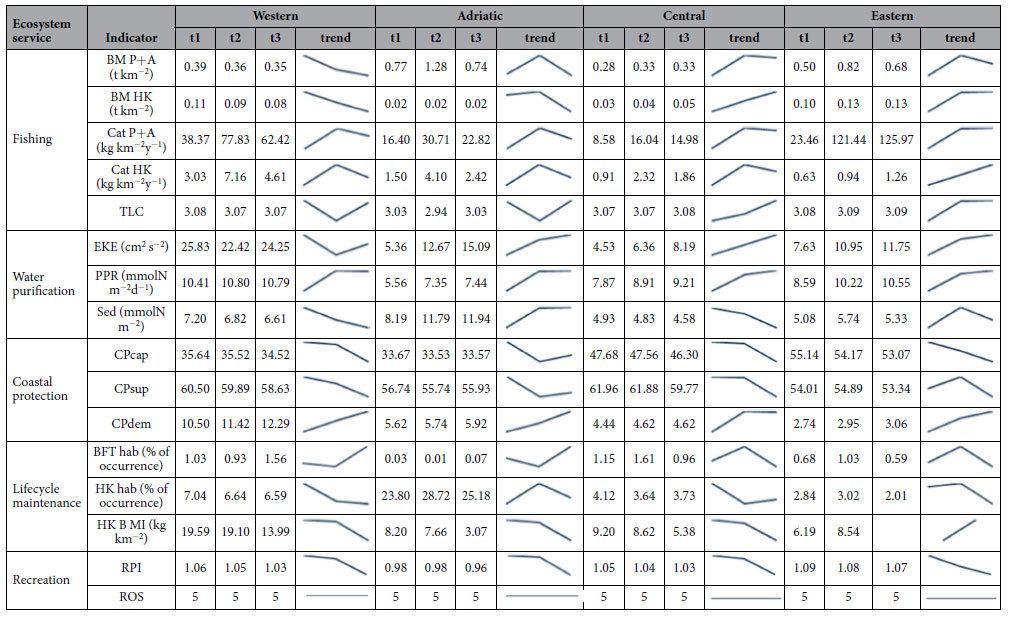
Results of all the analysed indicators aggregated per ecosystem service and per Mediterranean sub-basin.

**Table 3:** General trends of capacity (cap), flow and benefit (ben) of marine and coastal ecosystem services in the four sub-basins of the Mediterranean Sea.

Ecosystem service		Western	Adriatic	Central	Eastern
Food provisioning	cap	↘	↘	↗	↗
	flow	↗	↗	↗	↗
Water purification	cap	↘	↗	↗	↗
	flow	↔	↗	↗	↗
Coastal protection	cap	↘	↔	↘	↘
	flow	↘	↔	↘	↘
	ben	↗	↗	↗	↗
Lifecycle maintenance	cap	↔	↔	↘	↘
	flow	↘	↘	↘	
Recreation	cap	↔	↔	↔	↔
	flow	↔	↔	↔	↔
