# Supplementary material for: Ecosystem services sustainability in the Mediterranean Sea: assessment of status and trends using multiple modelling approaches
Source: Sci Rep. 2016 Sep 30;6:34162. doi: 10.1038/srep34162 (PMC5043175; doi:10.1038/srep34162)
Supplement: Supplementary Information [file srep34162-s1.pdf]

Supplementary Information

Ecosystem services sustainability in the Mediterranean Sea: assessment of status and trends using multiple modelling approaches

Camino Liquete[1]*, Chiara Piroddi[2], Diego Macias[1], Jean-Noël Druon[1], Grazia Zulian[1]

[1] European Commission, Joint Research Centre (JRC), Via Enrico Fermi 2749, 21027 Ispra, Italy

[2] Institute of Marine Science (ICM-CSIC), Barcelona, Spain

* Corresponding author: camino.liquete@gmail.com, +39 0332783799

SECTION I: THE CASCADE CONCEPTUAL FRAMEWORK FOR ECOSYSTEM SERVICES

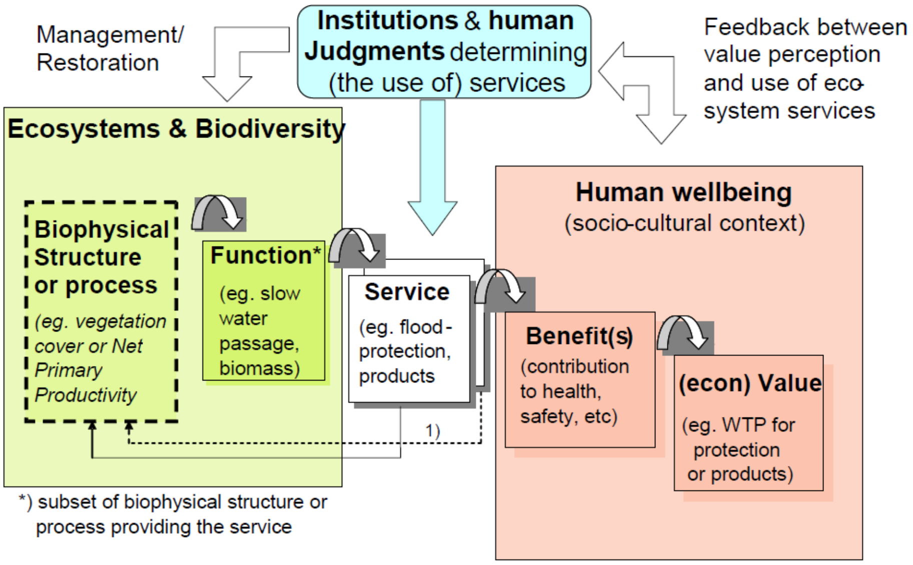

Supplementary Figure S1. The so-called cascade framework of ecosystem services, extracted from [1], adapted from [2].

To understand how benefits are provided to society and can be sustained in the long term, it is helpful to assess functions. Functions are defined in this context as the potential or capacity that ecosystems have to deliver a service and that depend on different ecological structures and processes. For example, primary production (=process) is needed to maintain a viable fish population (= function) which can be harvested to provide food (= service); the benefits can be manifold such as nutrition or employment by the fisheries industry, and the final value could be estimated through the market price of fish (although this price should be adjusted for any market imperfections, such as subsidies). In this paper we apply several modelling approaches to estimate the function (capacity), service (flow) and sometimes benefit of five marine and coastal ecosystem services.

SECTION II: ADDITIONAL DESCRIPTION OF THE MODELLING APPROACHES

1. Ecopath with Ecosim (EwE) model

1.1. Ecopath and the mass balanced approach

The *Ecopath* model is based on two main equations. In the first one (Eq.1), the biological production of a functional group is equal to the sum of fishing mortality, predation mortality, net migration, biomass accumulation, and other unexplained mortality.

$$(P/B)_i \cdot B_i = Y_i + \sum_j B_j \cdot (Q/B)_j \cdot DC_{ji} + E_i + BA_i + (P/B)_i \cdot B_i (1 - EE_i) \qquad \text{Eq.1}$$

where *(P/B)* is the production to biomass ratio for a certain functional group (*i*), *B*i is the biomass of a group (*i*), Y_i the total fishery catch rate of group (*i*), $(Q/B)_j$ is the consumption to biomass ratio for each predator (*j*), DC_{ji} is the proportion of the group (*i*) in the diet of predator (*j*), E_i is the net migration rate (emigration – immigration), BA_i is the biomass accumulation rate for the group (*i*), EE_i is the ecotrophic efficiency, and $(1 - EE_i)$ represents mortality other than predation and fishing.

In the second equation (Eq. 2), the consumption of a functional group is equal to the sum of production, respiration and unassimilated food.

Consumption = production + respiration +unassimilated food Eq.2

The implication of these two equations is that the model is mass-balanced; under this assumption, *Ecopath* uses and solves a system of linear equations (one for each functional group present in the system) estimating missing parameters. Therefore, the input parameters (B, P/B, Q/B, and DC) are entered first, and then the mass-balance in the model is ensured. To do so, we used the manual mass balanced procedure and we modified the model by adjusting the input parameters of those groups with EE > 1 [3].

1.2. Ecosim and the fitting procedure

Ecosim provides temporal simulations using the initial parameters of the *Ecopath* master equation. It works with a couple of differential equations to estimate biomass fluxes as follows:

$$dB_i/dt = g_i \sum Qji - \sum Qij + I_i - (M_i + F_i + e_i)B_i \qquad \text{Eq.3}$$

where dB_i/dt is the biomass growth rate of group (*i*) during the interval *dt*, g_i is the net growth efficiency (production/consumption ratio), I_i is the immigration rate, M_i and F_i are natural and fishing mortality rates of group (*i*), e_i is emigration rate[3,4]. The two summations estimate consumption rates, the first expressing the total consumption by group (i), and the second the predation by all predators on the same group (i). The consumption rates, *Q*ji, are calculated based on the 'foraging arena' concept, where Bi's are divided into vulnerable and invulnerable components[4], and it is the transfer rate (*v*ij) between these two components that determines if control is top-down (i.e. Lotka-Volterra) or bottom-up. When v is set high, e.g., of 100, predation control will be top down, conversely, if v is low (close to 1), replacement of depleted biomass from the invulnerable to the vulnerable part of the population will be

slow, and the amount that the predators consume will be largely determined by the low value of v, rather than by their own biomass. Thus, when v is low, control is bottom up. Using this procedure, *Ecosim* loads time series data on available biomasses of various functional groups over a particular historical period, along with estimates of catch and/or by-catch over that period. After such data are loaded, *Ecosim* runs several scenarios, using fishing effort and primary production as driving factors, and, per each scenario, a statistical measure of goodness of fit is generated. The primary production anomaly is a result of an *Ecosim* automated procedure that searches for time-series relative values (compare to the initial year) of annual production impacting biomasses of producer groups and their predators. This routine considers that if primary production concentration changes over time then the total amount of energy that enters in the ecosystem changes, causing a cascading-up effect that increases or decreases food availability through the ecosystem[5].

The goodness of fit is calculated as weighted sum of squared deviations (SS) of log 'observed' biomasses from log predicted biomasses. The fit that best represents the 'observed' data is chosen. The fit can be improved mainly by: 1) searching for changes in vulnerability estimates and/or by 2) searching, using a non-parametric routine incorporated in *Ecosim*, for time series values of annual relative primary productivity that may represent historical productivity 'regime shifts' impacting biomasses throughout the ecosystem. In addition to the nonlinear optimization routines, the fit to data can also be improved in a feedback-process by examining some of the crucial ecological parameters in the model (notably total mortality rates and the settings for top-down/bottom-up control) that are not set such that the model captures the observed trends over time adequately. The inclusion of time series data in EwE facilitates the exploration of policy options for ecosystem-based management of fisheries based on the assumption that if the model is capable of producing a reasonable fit, (i.e. fits that can be compared to those obtained from observed data) this indicates a capability or at least a potential to replicate the known history of the ecosystems and to explore policy scenarios[6].

1.3. Ecospace

The spatial-temporal dynamic module of the software, Ecospace, represents biomass (B) dynamics of marine species/functional groups over a two-dimensional space grid[6,7]. In the original version of Ecopace, the habitat allocated to each cell was considered as homogeneous and movement of biomass from a given cell to adjacent cells was assumed to vary based on habitat preferences and responses of organisms to depredation risk and feeding conditions. In the new Ecospace model, a habitat foraging capacity model has been implemented, allowing the probability of movement of organisms toward favorable habitats to be related to the suitability score of each cell, which can be define by combination of specific habitat attributes (e.g., depth, temperature, pH, bottom type)[8]. The suitability score of each cell affects the capacity of each functional group to forage in each cell, and thus links the habitat attributes to the trophic dynamics established in the food web based on the assumption that changes in habitat capacity will affect the cell-specific foraging arena available to a given functional group[8].

To parameterize the habitat capacity model in Ecospace, we linked the environmental preferences of each species/functional group of our Ecopath model to environmental spatial outputs obtained from a biogeochemical model using the new GIS linkage of Ecospace[9]. Environmental responses of each functional group to environmental parameters (e.g., depth, sea surface temperature and salinity) were extracted from the species distribution modelling framework Aquamaps[10] (www.aquamaps.org).

Supplementary Table S1. List of functional groups and fisheries and their abbreviations included in Ecopath and time-series data sources used in Ecosim. Also, functional groups and fisheries categories are the same in each Mediterranean sub-basin but their composition differ per area. This is not the case for highly migratory species (2. 'other cetaceans'; 5. 'sea turtles' and 6. 'large pelagics') that are allowed to move and feed in all the four areas[11]*.*

#	Functional groups/fisheries	Source of biomass time series	Source of catch time series
1	**Piscivorous cetaceans (PC):** *Delphinus delphis, Stenella coeruleoalba, Tursiops truncatus*		
2	**Others cetaceans (OC):** *Balaenoptera physalus, Globicephala melas, Grampus griseus, Physeter macrocephalus, Ziphius cavirostris*		
3	**Pinnipeds (PI):** *Monachus monachus*	[12–23]	
4	**Seabirds (SB):** *Calonectris diomedea, Hydrobates pelagicus melitensis, Larus michahellis, Larus audouinii, Larus genei, Larus melanocephalus, Phalacrocorax carbo, Puffinus yelkouan, Puffinus mauretanicus, Sterna nilotica, Sterna sandvicensis, Sterna caspia, Sterna hirundo, Sterna albifrons, Sterna bengalensis*		
5	**Sea turtles (ST):** *Caretta caretta, Chelonia mydas*	[24–36]	
6	**Large Pelagics (LP):** *Coryphaena hippurus, Tetrapturus belone, Thunnus alalunga, Thunnus thynnus, Xiphias gladius*	[37]	FishSTAT (www.fao.org) ; ICCAT (https://www.iccat.int)
7	**Medium pelagics (MP):** *Acanthocybium solandri, Alepes djedaba, Auxis rochei rochei, Auxis thazard thazard, Belone belone, Dicentrarchus punctatus, Euthynnus alletteratus, Katsuwonus pelamis, Lichia amia, Liza aurata, Orcynopsis unicolor, Pomatomus saltatrix, Sarda sarda, Scomber japonicus, Scomber scombrus, Scomberesox saurus saurus, Scomberomorus commerson, Seriola dumerili, Sphyraena sphyraena*		FishSTAT (www.fao.org)
8	**European pilchard (EP):** *Sardina pilchardus*	[38–53]	FishSTAT (www.fao.org)
9	**European anchovy /EA):** *Engraulis encrasicolus*	[38,39,43–53]	FishSTAT (www.fao.org)
10	**Other small pelagics (SP):** *Aphia minuta, Atherina hepsetus, Etrumeus sadina, Sardinella aurita, Spicara maena, Spicara smaris, Sprattus sprattus, Trachurus trachurus, Trachurus mediterraneus*		FishSTAT (www.fao.org)
11	**Large demersals (LD):** *Conger conger, Epinephelus aeneus, Epinephelus caninus, Epinephelus marginatus, Lophius piscatorius, Molva dypterygia, Muraena helena, Polyprion americanus*	International Bottom Trawl Survey in the Mediterranean (Medits database: http://www.sibm.it/SITO%20MEDITS/principaleprogramme.htm)	FishSTAT (www.fao.org)
12	**European hake (HK):** *Merluccius merluccius*		
13	**Medium demersals (MD):** *Argyrosomus regius, Balistes capriscus, Campogramma glaycos, Cepola macrophthalma, Chelidonichthys lucerna, Chelon labrosus, Dactylopterus volitans, Dentex dentex, Dentex macrophthalmus, Dicentrarchus labrax, Epigonus telescopus, Eutrigla gurnardus, Labrus Merula, Lagocephalus sceleratus, Lepidopus caudatus,Lithognathus mormyrus, Lophius budegassa, Mugil cephalus, Naucrates ductor, Pagellus bogaraveo, Pagrus pagrus, Phycis blennoides, Platichthys flesus, Plectorhinchus mediterraneus, Sarpa salpa, Saurida undosquamis, Sciaena umbra, Scophthalmus maximus, Scophthalmus rhombus, Scorpaena scrofa, Solea solea, Sparisoma cretense, Sparus aurata, Spondyliosoma cantharus, Trisopterus luscus, Umbrina canariensis, Umbrina cirrosa, Zeus faber*		
14	**Small demersals (SD):** *Atherina boyeri, Boops boops, Chelidonichthys cuculus, Dicologlossa cuneata, Diplodus annularis, Diplodus sargus sargus, Diplodus vulgaris, Gobius niger, Helicolenus dactylopterus, Lepidorhombus whiffiagonis, Merlangius merlangus, Mullus barbatus barbatus, Mullus surmuletus, Nemipterus randalli, Oblada melanura, Pagellus acarne, Pagellus erythrinus, Phycis phycis, Scorpaena porcus, Serranus cabrilla, Serranus scriba, Synodus saurus, Trachinus draco, Trisopterus minutes, Uranoscopus scaber, Xyrichtys novacula*		
15	**Deep fish (DF):** *Alepocephalus rostratus, Argyropelecus hemigymnus, Bathypterois mediterraneus, Benthocometes robustus, Benthosema glaciale, Brama brama, Caelorhynchus caelorhynchus, Caelorhynchus mediterraneus, Cataetyx laticeps, Ceratoscopelus maderensis, Chalinura mediterranea, Chauliodus sloani, Chlorophthalmus agassizii, Coryphaenoides guentheri, Cyclothone braueri, Diaphus metopoclampus, Epigonus constanciae, Epigonus denticulatus, Epigonus telescopus, Halosaurus ovenii, Helicolenus dactylopterus, Hoplostethus mediterraneus, Hygophum benoiti, Hymenocephalus italicus, Lampanyctus crocodilus, Lepidion lepidion, Lepidopus caudatus, Lepidorhombus whiffiagonis, Micromesistius poutassou, Mora moro, Nettastoma melanorum, Nezumia aequalis, Nezumia sclerorhynchus, Notacanthus bonapartei, Notolepis rissoi, Paralepis speciosa, Polyacanthonotus rissoanus, Stomias boa, Trachyrhynchus trachyrhynchus, Trachyscorpia cristulata echinata*	[54]	

16	**Sharks (SK):** Alopias superciliosus, Alopias vulpinus, Carcharias taurus, Carcharodon carcharias, Centrophorus granulosus, Centrophorus granulosus, Centroscymnus coelolepis, Cetorhinus maximus, Chimaera monstrosa, Dalatias licha, Etmopterus spinax, Galeorhinus galeus, Galeus melastomus, Heptranchias perlo, Hexanchus griseus, Isurus oxyrinchus, Lamna nasus, Mustelus mustelus, Oxinotus centrina, Prionace glauca, Scyliorhinus canicula, Sharks nei, Somniosus rostratus, Squalus acanthias, Squalus blainville	International Bottom Trawl Survey in the Mediterranean (Medits database: http://www.sibm.it/SITO%20MEDITS/principaleprogramme.htm)	FishSTAT (www.fao.org)
17	**Rays and skates (RS):** *Dasyatis pastinaca, Leucoraja naevus, Gymnura altavela, Mobula mobular, Myliobatis aquila, Rays and Skates nei, Raja asterias, Raja clavata, Raja montagui, Rhinobatos rhinobatos, Rostroraja alba*		
18	**Benthopelagic cephalopods (BPC):** *Alloteuthis media, Ancistroteuthis lichtensteini, Illex coindetii, Loligo vulgaris, Marine molluscs nei, Ostrea edulis, Sepia officinalis, Todarodes sagittatus*		
19	**Benthic cephalopods (BC)**: *Eledone cirrhosa, Eledone moschata, Marine molluscs nei, Octopus vulgaris*		
20	**Bivalves_gastropods (BG):** *Callista chione, Cerastoderma edule, Chamelea gallina, Crassostrea gigas, Donax vittatus, Littorina littorea, Marine molluscs nei, Mytilus galloprovincialis, Ostrea edulis, Pecten jacobaeus, Pecten maximus, Ruditapes decussatus, Venerupis pullastra, Venus verrucosa*		
21	**Crustaceans (CR):** *Aristaeomorpha foliacea, Aristeus antennatus, Carcinus aestuarii, Crangon crangon, Erugosquilla massavensis, Homarus gammarus, Maja squinado, Marine crustaceans nei, Marsupenaeus japonicus, Melicertus kerathurus, Metapenaeus monoceros, Nephrops norvegicus, Palaemon serratus, Palinurus elephas, Palinurus mauritanicus, Parapenaeus longirostris, Plesionika martia, Portunus pelagicus, Scyllarides latus, Squilla mantis*		
22	**Jellyfish (JF):** *Aequorea forskalea, Aurelia aurita, Pelagia noctiluca, Chrysaora hysoscella, Cotylorhiza tuberculata, Liriope tetraphylla, Mnemiopsis leidyi , Pleurobrachia rhodopis, Physalia physalis, Rhizostoma pulmo*		
23	**Benthos (BE):** nematodes, copepods (and naupliar stages), polychaetes, oligochaetes, isopods, cumaceans, amphipods, acarians, ostracods, oligochaetes, tanaidaceans, cnidarians, kinorhynchs, turbellarians, gastrotriches, nemerteans, bivalves, priapulids (including larvae), cladocerans, decapods (larvae) and echinoderms	[54]	
24	**Zooplankton (ZO):** meso and macro zooplankton (amphipods, copepods, cladocerans, euphasids, mysids, pteropods)		
25	**Phytoplankton (PH):** diatoms, dinoflagellates		
26	**Seagrass (SE):** *Cymodocea nodosa, Posidonia oceanica, Zoostera marina, Zoostera noltii*		
27	**Detritus (DE)**		
28	**Discards (DI)**		
29	**Trawlers (TR)**		
30	**Purse seiners (PS)**		
31	**Long liners (LL)**		
32	**Artisanal fisheries (AR)**		
33	**Recreational fisheries (RC)**		

Supplementary Table S2. Initial (grey cells) and output (white cells) parameters of the Mediterranean marine ecosystem for the 1950s period. Functional groups/species of the model are separated following the four Mediterranean sub-basins: Western (W); Adriatic (A); Ionian and Central Mediterranean (I); Aegean and Levantine (E).

#	Group name	Trophic level	Habitat area (fraction)	Biomass in habitat area (t/km²)	Biomass (t/km²)	Production / biomass (/year)	Consumption / biomass (/year)	Ecotrophic efficiency	Production/ consumption
1	Piscivores cetaceans W	4.19	0.33	0.01	0.00	0.08	25.84	0.97	0.00
2	Others cetaceans	3.53	1.00	0.07	0.07	0.05	8.29	0.07	0.01
3	Pinnipeds W	4.20	0.33	0.00	0.00	0.08	13.15	0.90	0.01
4	Seabirds W	3.09	0.33	0.00	0.00	5.33	73.09	0.01	0.07
5	Sea turtles	2.68	1.00	0.02	0.02	0.19	2.78	0.14	0.07
6	Large Pelagics	3.94	1.00	0.44	0.44	0.35	2.50	0.04	0.14
7	Medium pelagics W	3.28	0.33	0.56	0.18	0.75	4.94	0.85	0.15
8	European pilchard W	3.13	0.33	0.55	0.18	0.99	8.45	1.00	0.12
9	European anchovy W	3.25	0.33	0.67	0.22	0.87	7.95	0.90	0.11
10	Other small pelagics W	3.14	0.33	0.36	0.12	0.75	6.63	0.90	0.11
11	Large demersals W	3.68	0.33	0.24	0.08	0.87	3.06	0.87	0.28
12	European hake W	3.81	0.33	0.28	0.09	0.60	2.80	0.91	0.21
13	Medium demersals W	2.94	0.33	0.79	0.26	0.70	6.40	0.92	0.11
14	Small demersals W	3.03	0.33	0.38	0.13	1.57	6.87	0.98	0.23
15	Deep fish W	2.97	0.33	0.85	0.28	0.70	3.50	0.99	0.20
16	Sharks W	3.85	0.33	0.36	0.12	0.42	3.48	0.10	0.12
17	Rays and skates W	3.34	0.33	0.28	0.09	0.80	3.67	0.83	0.22
18	Benthopelagic cephalopods W	3.69	0.33	0.32	0.11	2.00	9.00	0.96	0.22
19	Benthic cephalopods W	3.44	0.33	0.56	0.18	2.10	7.00	0.86	0.30
20	Bivalves_gastropods W	2.01	0.33	1.00	0.33	1.30	5.00	0.94	0.26
21	Crustaceans W	2.79	0.33	0.99	0.33	3.50	12.00	0.97	0.29
22	Jellyfish W	3.08	0.33	0.33	0.11	13.87	50.48	0.42	0.27
23	Benthos W	2.02	0.33	16.22	5.39	2.50	9.04	0.33	0.28
24	Zooplankton W	2.25	0.33	7.76	2.58	30.60	102.00	0.81	0.30
25	Phytoplankton W	1.00	0.33	18.40	6.11	197.00	--	0.18	--
26	Seagrass W	1.00	0.33	16.70	5.55	5.94	--	0.16	--
27	Piscivores cetaceans A	4.16	0.05	0.00	0.00	0.08	25.84	0.90	0.00
28	Pinnipeds A	4.19	0.05	0.00	0.00	0.08	13.15	0.55	0.01
29	Seabirds A	3.03	0.05	0.00	0.00	4.61	69.34	0.16	0.07
30	Medium Pelagics A	3.26	0.05	0.88	0.05	0.92	6.76	0.89	0.14
31	European pilchard A	3.00	0.05	4.32	0.23	0.80	9.19	0.31	0.09
32	European anchovy A	3.11	0.05	2.60	0.14	0.85	11.02	0.75	0.08
33	Other small pelagics A	3.02	0.05	0.53	0.03	1.00	11.29	0.48	0.09
34	Large demersals A	3.63	0.05	0.20	0.01	0.90	5.14	0.72	0.18
35	European hake A	3.86	0.05	0.28	0.01	0.40	1.85	0.79	0.22
36	Medium demersals A	2.96	0.05	0.39	0.02	1.10	5.57	0.99	0.20
37	Small demersals A	2.96	0.05	0.32	0.02	1.50	8.02	0.97	0.19
38	Deep fish A	2.88	0.05	0.61	0.03	0.70	3.50	0.98	0.20
39	Sharks A	3.79	0.05	0.10	0.01	0.50	4.00	0.26	0.13
40	Rays and skates A	3.41	0.05	0.12	0.01	0.64	4.10	0.77	0.16
41	Benthopelagic cephalopods A	3.58	0.05	0.22	0.01	2.70	9.00	0.88	0.30
42	Benthic cephalopods A	3.45	0.05	0.33	0.02	2.10	7.00	0.85	0.30
43	Bivalves_gastropods A	2.05	0.05	0.95	0.05	1.30	5.00	0.99	0.26
44	Crustaceans A	2.76	0.05	0.80	0.04	3.50	12.00	0.99	0.29
45	Jellyfish A	3.14	0.05	2.27	0.12	14.60	50.48	0.94	0.29
46	Benthos A	2.02	0.05	68.24	3.64	1.31	6.71	0.18	0.20
47	Zooplankton A	2.11	0.05	5.79	0.31	37.85	126.17	0.97	0.30
48	Phytoplankton A	1.00	0.05	15.00	0.80	140.00	--	0.33	--
49	Seagrass A	1.00	0.05	2.68	0.14	4.02	--	0.50	--
50	Piscivores cetaceans I	4.13	0.30	0.00	0.00	0.08	25.84	0.77	0.00
51	Pinnipeds I	4.16	0.30	0.00	0.00	0.08	13.15	0.35	0.01
52	Seabirds I	3.11	0.30	0.00	0.00	4.60	105.43	0.07	0.04

53	Medium Pelagics I	3.20	0.30	0.38	0.11	0.70	7.70	0.96	0.09
54	European pilchard I	3.02	0.30	0.48	0.14	0.94	8.68	0.97	0.11
55	European anchovy I	3.14	0.30	0.53	0.16	0.91	12.30	0.86	0.07
56	Other small pelagics I	3.04	0.30	0.28	0.08	0.86	8.36	0.95	0.10
57	Large demersals I	3.66	0.30	0.20	0.06	0.65	2.85	0.93	0.23
58	European hake I	3.86	0.30	0.13	0.04	0.65	3.40	0.96	0.19
59	Medium demersals I	2.89	0.30	0.65	0.20	0.90	8.13	0.82	0.11
60	Small demersals I	2.93	0.30	0.34	0.10	1.10	6.38	0.98	0.17
61	Deep fish I	2.80	0.30	0.59	0.18	0.70	3.50	0.81	0.20
62	Sharks I	3.72	0.30	0.24	0.07	0.41	4.33	0.10	0.09
63	Rays and skates I	3.27	0.30	0.24	0.07	0.60	3.00	0.76	0.20
64	Benthopelagic cephalopods I	3.53	0.30	0.17	0.05	2.70	9.00	0.93	0.30
65	Benthic cephalopods I	3.42	0.30	0.33	0.10	2.10	7.00	0.95	0.30
66	Bivalves_gastropods I	2.01	0.30	0.70	0.21	1.30	5.00	0.95	0.26
67	Crustaceans I	2.63	0.30	0.63	0.19	3.45	12.00	0.97	0.29
68	Jellyfish I	3.10	0.30	0.17	0.05	11.10	35.90	0.87	0.31
69	Benthos I	2.01	0.30	11.74	3.52	2.75	22.00	0.29	0.13
70	Zooplankton I	2.14	0.30	3.63	1.09	38.44	128.12	0.57	0.30
71	Phytoplankton I	1.00	0.30	7.60	2.28	61.80	--	0.88	--
72	Seagrass I	1.00	0.30	16.00	4.79	2.59	--	0.64	--
73	Piscivores cetaceans E	4.12	0.31	0.00	0.00	0.08	25.84	0.76	0.00
74	Pinnipeds E	4.11	0.31	0.00	0.00	0.08	13.15	0.31	0.01
75	Seabirds E	3.12	0.31	0.00	0.00	4.78	111.61	0.00	0.04
76	Medium Pelagics E	3.23	0.31	0.61	0.19	0.80	4.79	0.92	0.17
77	European pilchard E	3.02	0.31	0.48	0.15	0.95	9.49	0.99	0.10
78	European anchovy E	3.14	0.31	0.87	0.27	0.90	5.20	0.92	0.17
79	Other small pelagics E	2.89	0.31	0.48	0.15	0.95	8.23	0.91	0.12
80	Large demersals E	3.57	0.31	0.18	0.06	0.70	4.35	0.94	0.16
81	European hake E	3.79	0.31	0.28	0.09	0.60	5.26	0.92	0.11
82	Medium demersals E	2.87	0.31	0.40	0.12	1.00	9.09	0.91	0.11
83	Small demersals E	2.95	0.31	0.36	0.11	1.10	7.64	0.99	0.14
84	Deep fish E	2.90	0.31	0.42	0.13	0.70	3.50	0.94	0.20
85	Skarks E	3.70	0.31	0.20	0.06	0.50	5.16	0.10	0.10
86	Rays and skates E	3.38	0.31	0.18	0.06	0.70	4.07	0.93	0.17
87	Benthopelagic cephalopods E	3.55	0.31	0.13	0.04	2.70	9.00	0.92	0.30
88	Benthic cephalopods E	3.36	0.31	0.32	0.10	2.10	7.00	0.96	0.30
89	Bivalves_gastropods E	2.01	0.31	0.62	0.19	1.30	5.00	0.98	0.26
90	Crustaceans E	2.64	0.31	0.56	0.17	3.50	12.00	0.98	0.29
91	Jellyfish E	3.25	0.31	0.16	0.05	4.84	15.00	0.75	0.32
92	Benthos E	2.02	0.31	9.83	3.10	2.64	16.13	0.32	0.16
93	Zooplankton E	2.14	0.31	3.59	1.13	38.80	129.33	0.55	0.30
94	Phytoplankton E	1.00	0.31	8.83	2.78	70.00	--	0.66	--
95	Seagrass E	1.00	0.31	15.00	4.72	2.69	--	0.40	--
96	Discards W	1.00	0.33	0.02	0.01	--	--	0.02	--
97	Detritus W	1.00	0.33	32.01	10.63	--	--	0.04	--
98	Discards A	1.00	0.05	0.01	0.00	--	--	0.11	--
99	Detritus A	1.00	0.05	19.73	1.05	--	--	0.25	--
100	Discards I	1.00	0.30	0.01	0.00	--	--	0.27	--
101	Detritus I	1.00	0.30	14.78	4.43	--	--	0.59	--
102	Discards E	1.00	0.31	0.01	0.00	--	--	0.13	--
103	Detritus E	1.00	0.31	14.74	4.64	--	--	0.29	--

2. Coastal protection model

2.1. New indicators

The main updates from the first version of the coastal protection model[55] to this new version are: (a) we avoid extrapolating data to fill gaps, instead we keep blanks and ignore them in the estimation of indicators, (b) we exclude the experts' weighting factors previously applied to the variables of CPcap and CPexp, (c) we update most of the data sources and include a temporal trend, (d) we avoid ranking and cross-tabulating CPcap and CPexp to integrate them and use the new CPsup instead, (e) we exclude the presence of infrastructures from CPdem since it can be redundant with the other variables and the data set seemed to be country-biased. As a result, now all the indicators have continuous values and are calculated for the years 1990, 2000 and 2010. The new indicators are estimated as follows:

$$\text{CPcap} = (\text{geo} + \text{slo} + \text{sea} + \text{lan}) \,/\, \sum \text{no. of variables}$$

$$\text{CPexp} = (\text{wav} + \text{sur} + \text{lev} - \text{tid}) \,/\, \sum \text{no. of variables}$$

$$\text{CPsup} = \text{CPcap} - \text{CPexp}$$

$$\text{CPdem} = 0.4\ \text{pop} + 0.4\ \text{art} + 0.2\ \text{cul}$$

The variables and data sources used to construct these indicators are summarised in Table S3 and explained below.

Supplementary Table S3. List of variables and data sources used in the coastal protection model.

Variable	Acronym	Selected value & units	Temporal coverage	Data source
Coastal geomorphology	geo	Weighted average per length (protection score)	1990, 2000, 2010	EUROSION Coastal Erosion Layer[56]
Slope	slo	Average slope (%)	static	GTOPO30 digital elevation model (https://lta.cr.usgs.gov/GTOPO30)
Seabed habitats	sea	Weighted average per area (protection score)	static	EUSeaMap products (http://www.emodnet.eu/seabed-habitats) and EUNIS marine compilation[57]
Land use/land cover	lan	Weighted average per area (protection score)	1990, 2000, 2010	CLC 1990, 2000 and 2006 from EEA (http://www.eea.europa.eu/data-and-maps)
Wave regime	wav	Average of the maximum individual wave height (m)	1990, 2000, 2010	ERA-20C data set from ECMWF (http://www.ecmwf.int/en/research/climate-reanalysis/era-20c)
Storm surge	sur	Weighted average per length of a 1 in 100 years surge wave height (m)	static	Global storm surge height data from the DIVA database (http://www.diva-model.net/)
Sea level rise	lev	Average sea level anomaly over a 20 yr period (m)	static	Sea level anomalies from AVISO+ satellite altimetry data (http://aviso.altimetry.fr/index.php?id=1526)

Tidal amplitude	tid	Average tidal amplitude (cm)	static	AVISO+ FES2012 data set (http://aviso.altimetry.fr/index.php?id=1279)
Population	pop	Sum of total population (inhab)	1990, 2000, 2010	GEOSTAT population grid 2011[58] and regional population change[59]
Artificial surface	art	Density in percentage of area (%)	1990, 2000, 2010	CLC 1990, 2000 and 2006 from EEA (http://www.eea.europa.eu/data-and-maps)
Main cultural sites	cul	Sum of sites (no.)	static	UNESCO World Heritage List (http://whc.unesco.org)

2.2. Data sources and processing

2.2.1. Coastal geomorphology

EUROSION Coastal Erosion database[56] includes morpho-sedimentological, erosion trends, geological patterns and coastal defence works of the European coastline. We use the fields CEMO (Coastal Erosion MOrpho-sedimentological code) and CEEV (Coastal Erosion EVolutionary trend). Each coastal feature (CEMO) is scored for their protection capacity following table S2. This is based on a coastal experts' questionnaire run in 2011-2012[55]. The level of protection ranges from 1 (very low) to 5 (very high), with 0 meaning no data.

The EUROSION Coastal Erosion Layer classifies most of the EU shoreline with data that can be assumed to be representative of the year 2000, and it also includes the 1990 CORINE Coastal Erosion database, which covered EU-15. To characterise the year 2010 and the missing sections of the 1990 database, the coastal evolutionary trends (erosion, aggradation, stability) of the year 2000 have been used as weighting factors to be multiplied by the level of protection. It is assumed that a shoreline undergoing erosion in 2000 must have been in a better state in 1990 (i.e. its level of protection of 2000 is multiplied by a weighting factor of 1.1 or 1.2) and will be in a worn state in 2010 (weighting factor of 0.8 or 0.9), and vice versa for aggradation. In case of stable shoreline or lack of data the level of protection of 2000 is not modified.

Supplementary Table S4: Level of coastal protection provided by different coastal features and habitats. This ranking is used to transform the qualitative data of the model into the quantitative indicators.

Protection capacity	***Coastal morpho-sedimentology***	***Seabed or coastal habitat***	***Land cover***
5	Rocks, hard cliffs, developed beaches (from fine sand to pebbles)	Rock, coastal dunes & shingle	Beaches, dunes, sands
4	Conglomerates, eroded cliffs, small beaches, rocks or cliffs with small beaches	Protective communities (e.g. coralligenous, seagrass, kelp, maerl, macrophytes, lagoons)	Forests, transitional woodland-shrub
3	Soft non-cohesive sediments, muddy sediments, artificial beaches, soft strands with rocks	Coarse or mixed sediments, high energy sand	Wetlands and lagoons, scrub and/or herbaceous vegetation associations, agro-forestry areas, fruit and olive plantations
2	Estuaries, pond or lake shore type, soft strands of heterogeneous category	Sand, undefined 'sediment', detritic or seabed	Artificial vegetated areas, estuaries, vineyards, pastures, complex cultivation patterns, agricultural areas with natural vegetation

1	Reclaimed coastal areas	Mud	Arable land, annual crops, open spaces with little or no vegetation (excluding beaches & dunes), inland waters
0	Harbour areas, coastal constructions, artificial shoreline, dikes, unclassified	Deep sea or bathyal, unclassified	Artificial surfaces, unclassified

2.2.2. Slope

GTOPO30 (https://lta.cr.usgs.gov/GTOPO30) is a global digital elevation model with a horizontal grid spacing of 30 arc-seconds (approximately 1 kilometre) covering the full range of latitudes. Data is available from the U.S. Geological Survey (http://eros.usgs.gov/find-data). With this data set, we calculate the average slope per coastal operational unit.

2.2.3. Seabed habitats

The EMODnet SeaBed habitats portal hosts the EUSeaMap products (http://www.emodnet.eu/seabed-habitats). We extract the Western Mediterranean official 2012 seabed habitat map from the EUSeaMap 2014-2015 products. Other draft interim products like the Adriatic or Eastern Mediterranean seabed habitat maps are not publicly available yet. We complete the Mediterranean coverage with the EUNIS marine compilation[57] at the maximum possible detail (both in resolution and EUNIS hierarchy level). This compilation is available in the EMIS Marine Maps Platform (http://139.191.12.164/emis/index.py). After the integration of these data sets we score the values in terms of level of protection as in Table S4. Still, there are some gaps in the Croatian coastal waters.

2.2.4. Land use/land cover

We use the European Corine Land Cover (CLC) maps for 1990, 2000 and 2006 at 100 m resolution. We complete the CLC 1990 and 2006 layers where needed with the 2000 data set to get a full EU coverage for the three years. We had to assume that 2006 can be representative for the year 2010 in our analysis. The reclassification of land cover data into level of protection, based on expert opinion, friction coefficients and the questionnaire run in 2011-2012, is shown in Table S4.

2.2.5. Wave regime

We extract monthly data of maximum individual wave height from the ERA-20C reanalysis of the 20th century from the European Centre for Medium-Range Weather Forecasts (ECMWF) (http://www.ecmwf.int/en/research/climate-reanalysis/era-20c). In ERA-20C, a coupled atmosphere, land-surface & ocean-waves model is used to reanalyse the weather, by assimilating surface observations. We estimate an overall mean of five years around 1990, 2000 and 2010 as a representative value of those dates. The inter-annual variability is very low. Finally we calculate the average value of maximum individual wave height per operational unit.

2.2.6. Storm surge

We use the same data set as in 2013: the height of a surge wave with return period of 100 years from the Dynamic Interactive Vulnerability Assessment (DIVA) database (http://www.diva-model.net/, the database is no longer available through the internet). However, this time we estimate as an input variable the weighted average of surge wave height per length of shoreline, instead of the simple average. The height is expressed as meters above mean sea level. Calculation and original data (tidal levels, barometric pressures, wind speeds and sea bed slopes) are explained in [60].

2.2.7. Sea level rise

We extract the seasonal climatologies (1993-2014) of the sea level anomalies from AVISO+ satellite altimetry data. The anomalies are sea surface heights computed with respect to a twenty-year mean[61] which is a significant upgrade from the version used in [55], based on a seven years period. The altimeter products are produced by Ssalto/Duacs and distributed by Aviso, with support from Cnes (http://www.aviso.altimetry.fr/duacs/).

2.2.8. Tidal amplitude

We extract the amplitude of the main semi-diurnal component of the tide or principal lunar tide (wave M2) from the AVISO+ FES2012 data set (http://aviso.altimetry.fr/index.php?id=1279). The FES (Finite Element Solution) tide model is a combined product based on an hydrodynamic model which assimilate altimeter data. This version from 2012, compared with the previously used FES2004, improves particularly in coastal and shelf regions and has a better resolution[62]. FES2012 takes advantage of longer altimeter time series, improved modelling and data assimilation techniques, and more accurate ocean bathymetry. FES2012 was produced by Noveltis, Legos and CLS Space Oceanography Division and distributed by Aviso, with support from Cnes (http://www.aviso.altimetry.fr/).

2.2.9. Population

Although there are some spatially disaggregated population grids for Europe, there is no consistent data source that covers the period 1990-2010 and allows for a continuous analysis or comparison. Due to this limitation, we decide to estimate population trends from (1) the GEOSTAT Grid 2011 that reflects the number of persons at their usual place of residence per square kilometre for the census reference year 2011[58], and (2) the regional demographic statistics from EUROSTAT, in particular the annual data on population density (population per square kilometre) at NUTS-3 level[59] that shows the population temporal trends.

We estimate the population change between 2011 (assumed to be representative of 2010), 2000 and 1990 at NUTS-3 level. When data on the past years (1990 or 2000) is not available we use the closest possible value to estimate an annual trend and extrapolate linearly until the required year. Then we apply the regional trends to the GEOSTAT Grid 2011 to get an approximation of the inhabitants per square kilometre in 1990, 2000 and 2010. Our final variable is the sum of total population living within each coastal operational unit.

2.2.10. Artificial surface

Data sources and processing are similar to the coastal land cover (above). This time we extract the artificial surface from the CLC maps and estimate the percentage of artificial surface in each operational unit. The CLC artificial surface includes the major infrastructures.

2.2.11. Main cultural sites

The sites of outstanding universal value are included in the UNESCO World Heritage List. At present there are 1031 cultural, natural or mixed sites in the World Heritage List (compared with the 936 present during the previous analysis[55]), of which 91 lay in the study area with an occurrence between 0 and 4 per operational unit. Data is extracted from the UNESCO/WHC home page (http://whc.unesco.org, Copyright © 1992 - 2015 UNESCO/World Heritage Centre, all rights reserved).

3. Fish habitat models

We use two different ecological niche models centred on the reproductive aspects of fish. The first model analyses the favourable feeding conditions of the year-0 group (or 0 to 15 cm total length) European hake, *Merluccius merluccius*. The second model focuses on the preferred spawning conditions of the Atlantic bluefin tuna, *Thunnus thynnus*. The main steps of the modelling development (fully described in [63,64] respectively) are:

1. The analysis of the main ecological traits of the species and their linkage to the environment. It was performed using presence-only (tuna) or abundance (hake) data and complemented, when necessary, with the scientific literature.
    a. Hake nurseries: Druon et al.[63] analysed the characteristics (e.g. growth rates) of first life stages of hake when settled at seabed to define the main period under which the recruits were influenced by the environment. It was found that the main period where the preferential habitat for recruits should be integrated was from February to June, five months prior sampling date. Main sources of information related to distribution, abundance and size of the year-0 group hake in the Mediterranean Sea were extracted from the MEDITS International Bottom Trawl Survey[65].
    b. Bluefin tuna spawning: Druon et al.[64] analysed the suitable spawning conditions of Atlantic bluefin tuna concluding that they are traced by favourable meso-scale oceanographic features within a thermally stratified upper waters which favour larvae retention, feeding and transport. Specific ranges of surface chlorophyll-a concentrations, sea surface temperature, sea surface height anomaly and monthly increase of surface temperature were used to track the potential spawning habitat. Bluefin tuna spawning indeed notably occurs in warm waters of the Mediterranean Sea from mid-May to early July, preferably in areas with well-stratified surface waters and lower productive waters compared to where adults generally feed (see details in [64]).
2. Data collection and processing

a. Hake nurseries: Based on a specific processing of selected MEDITS data, Druon et al.[63] calculated a biomass index of hake recruits for each haul. The main environmental variables used for the hake model were: surface chlorophyll-a concentrations originating from the MODIS Aqua and SeaWiFS satellite sensors (http://oceancolor.gsfc.nasa.gov/) and processed at daily time scale also to identify chlorophyll fronts (horizontal gradient); monthly mean sea surface and bottom current velocity as well as sea surface and bottom temperature from the Mediterranean Forecasting System of MyOcean (www.MyOcean.eu); and bathymetry data from GEBCO (http://www.gebco.net).
b. Bluefin tuna spawning: The input data for the tuna spawning habitat model included[64]: presence data with precise geolocation from commercial fisheries, scientific surveys and recreational fisheries with for most of the data information on fish weight (or alternatively length); daily chlorophyll concentrations and fronts from MODIS Aqua; and sea surface height anomaly, current velocity and temperature derived from the Mediterranean hydrodynamic model of MyOcean Consortium (NEMO-OPA v3.2).

3. For both models, a cluster analysis derived on the retained set of environmental variables at the location and time of the species observations was used to define the relevant environmental thresholds that separate favourable from unfavourable habitats. Results showed to be consistent with the regional knowledge and literature information.

4. Recreation model

The final Recreation Potential Index (RPI) presented in this study is the combination of the normalised terrestrial and marine information ($RPI = RPI_{terrestrial} + RPI_{marine}$) reported at the level of Local Administrative Units (LAUs). It is a dimensionless indicator that ranges from 0 to 1.62. The final Recreation Opportunity Spectrum (ROS) is a categorical indicator with values that range between 1 and 9 as described in Table S5. In this case, the final results per LAU correspond to the mode or most frequent value of ROS within each reporting unit.

4.1. Update of the terrestrial model

There are a few updates in the new run of this model compared with the methodology published in [66,67]:

- Natural/pristine ecosystems are rare in Europe and it is difficult to reach a common definition. For the application of the model to the coastal area the concept of naturalness was replaced with the concept of “suitability of land to support nature-based recreation”.
- RPI includes the presence of natural riparian zones and of green urban areas.
- The database of Natural Protected areas has been updated with Natura 2000 sites; nationally designated areas (CDDA) 2014 (http://www.eea.europa.eu/data-and-maps/data/nationally-designated-areas-national-cdda-9#tab-gis-data); and the World Database on Protected Areas (WDPA) (http://www.protectedplanet.net/).

- The natural protected areas were classified, where possible, according to IUNC categories (http://www.iucn.org/about/work/programmes/gpap_home/gpap_quality/gpap_pacategories/) and according to the management objectives (see [68]).
- Two of the cascade indicators (RPI and ROS) are estimated for the years 1990, 2000 and 2006 (due to the lack of newer consistent land cover data sets, 2006 is considered as representative for the year 2010).
- The analysis is extended to Croatia.
- All the results of the two dimensionless indicators are normalized (based on minimum and maximum values) for the new study area: the Mediterranean region.
- The classes and cross-tabulations for the remoteness, proximity and ROS are specified in table S5.

Supplementary Table S5: Ranking of the proximity and Recreation Opportunity Spectrum classes.

		Distance from roads (km)				
		<0.5	0.5-1	1-5	5-10	> 10
Distance from urban (km)	<0.5	1	1	2	3	4
	0.5-1	1	1	2	3	4
	1-5	2	2	2	4	5
	5-10	3	3	4	5	5
	> 10	3	4	4	5	5

Proximity classes

near	**1**
	**2**
proximal	**3**
far	**4**
	**5**

		RPI classes			
		very low	low	high	very high
		1	2	3	4
Proximity classes	5	1	1	4	7
	4	1	4	4	7
	3	2	2	8	8
	2	3	5	5	9
	1	3	6	6	9

ROS classes

low provision	far	**1**
	proximal	**2**
	near	**3**
medium	far	**4**
	proximal	**5**
	near	**6**
high	far	**7**
	proximal	**8**
	near	**9**

4.2. Data sources and processing for the new marine component of RPI

4.2.1. Coastal geomorphology

The morpho-sedimentology of the shoreline (rocky coasts, beaches, muddy coasts, etc.) is a crucial factor to determine potential recreational activities and people's attraction to the coast. In this model the EUROSION Coastal Erosion Layer[56] is used to locate the different coastal morphological features. Each feature is scored for their suitability for recreation following Table S6. The location of Blue Flag beaches

(http://www.blueflag.global/) has been used to add some beach category (with maximum suitability for recreation) where the data coverage was very poor, i.e. in Croatia and Cyprus.

The Coastal Erosion Layer classifies most of the EU shoreline with data that can be assumed to be representative of the year 2000. The EUROSION project revised also the 1990 CORINE Coastal Erosion database, which covered EU-15. For the year 2010 and the missing sections of the 1990 CORINE Coastal Erosion database, the coastal evolutionary trends (erosion, aggradation, stability) of the year 2000 have been used as weighting factors to be multiplied by the recreation potential. It is assumed, for instance, that a beach undergoing erosion in 2000 must have been better for recreation in 1990 (i.e. its recreation potential of 2000 is multiplied by a weighting factor of 1.1 or 1.2) and will be worst in 2010 (weighting factor of 0.8 or 0.9), and vice versa for aggradation. It is assumed that a shoreline undergoing erosion in 2000 must have been in a better state in 1990 (i.e. its level of protection of 2000 is multiplied by a weighting factor of 1.1 or 1.2) and will be in a worn state in 2010 (weighting factor of 0.8 or 0.9), and vice versa for aggradation. In case of stable shoreline or lack of data the recreation potential of 2000 is not modified. Finally, the results are normalised linearly in a scale from 0 (null) to 1 (maximum).

Supplementary Table S6: Level of recreation potential provided by different coastal features. This ranking is used to transform the qualitative data of the model into the quantitative indicators.

Recreation potential	***Coastal morpho-sedimentology***
1	developed beaches, small beaches, small beaches separated by rocky capes, artificial beaches
0.8	Coastlines made of soft non-cohesive sediments, soft strands with beach-rock
0.6	Rocks, hard cliffs, pond or lake shore type, soft strands with rocky intertidal flat
0.5	Conglomerates, eroded cliffs
0.3	Soft strands of heterogeneous category or of unknown category grain size
0	Strands made of muddy sediments, estuaries, harbour areas, coastal embankments for construction, polders, mine-waste sediments, artificial shoreline, dikes, unclassified

4.2.2. Ecological status or potential

The data for characterizing the ecological status of water bodies have been extracted from the WISE2 databases. These databases contain data from River Basin Management Plans reported by EU Member States according to article 13 of the Water Framework Directive (WFD). WISE2 was compiled by the EEA in 2012 covering data from 2004 to 2009 (http://www.eea.europa.eu/data-and-maps/data/wise_wfd). The data is not made available for public download since it can be considered provisional and incomplete on some issues, but a more developed update is expected for 2016.

The original tables related to surface water bodies contain information about the ecological status or potential of EU coastal and transitional waters. The ecological status can be reported as high, good, moderate, poor or bad, with specific thresholds defined in the WFD (European Commission, Directive

2000/60/EC) and during the intercalibration exercise. For this analysis, we located the centroid of each water body with valid data on ecological status (3061 points in total) and assigned them a circular area around the centroid equal to the reported area of each water body. Then, we reclassified the reporting values as follows: high & good ecological status = score 1 for recreation (positive effect); moderate ecological status and No Data = score 0 for recreation (no effect); poor & bad ecological status = score -1 for recreation (negative effect). Large data gaps are found in Italy, Croatia and the French Aquitaine. Once the data are aggregated per coastal units, the scale is transformed into 0-1. Since we only have a punctual set of information, we consider these data static for our model.

4.2.3. Water clarity

Water clarity is usually characterized by the diffuse attenuation coefficient for downward irradiance at 490 nm or visible light in the blue-green (Kd490). Kd490 is a coefficient describing the exponential decay of the optical depth and has units of 1/m. Large Kd490 values (i.e. large attenuation with depth) indicate poor water clarity. Water turbidity increases with the presence of scattering particles in the water column, either organic or inorganic, and it is assumed to affect tourism and recreation.

Modelled data of Kd490 derived from different satellite sensors are available through the NASA's OceanColor Web (http://oceancolor.gsfc.nasa.gov/cms/). We selected the annual composites of Modis Aqua for this analysis and treated them with the help of Marine Geospatial Ecology Tools[69]. Data have around 4 km resolution and cover from 2002 to 2014. We generated the closest data layers representative of the years 2000 (the average of 2002 & 2003) and 2010 (the average of 2009-2011). Even if there are some sensors that retrieved older data (e.g. CZCS from 1978 to 1986), they were tested and found incompatible with the modern MODIS data series. Thus, the layer representative of the year 2000 was also applied for 1990. In all cases Kd490 ranged between 0.01 and 6.4 m^{-1}. Finally, the results are ranked into 11 classes in a scale from 0 (maximum Kd490, null for recreation) to 1 (minimum Kd490, maximum for recreation).

SECTION III: ADDITIONAL MAPS OF RESULTS

Figures 1-5 of the main manuscript show examples that illustrate the results, in particular the most recent quantification of each indicator. In this section of the Supplementary Information we include the additional time periods per indicator and the spatial changes from the first to the last stage. The only exception is the spatial analysis of food provisioning which is still an ongoing work and results are not definitive yet.

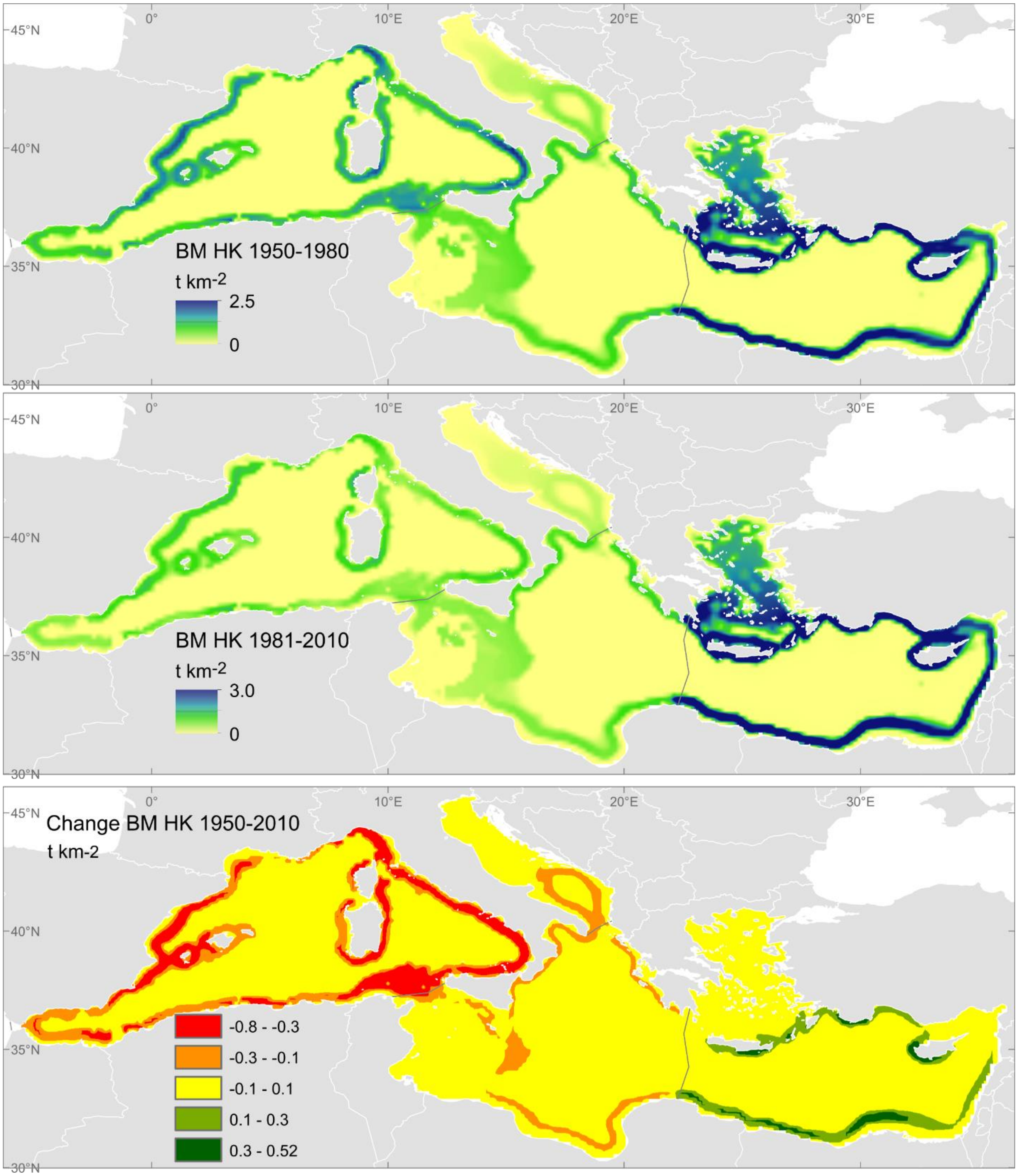

Supplementary Figure S2. Distribution through time of the biomass of European hake (BM HK), indicator of the capacity of food provisioning. Despite the fact that spatial analysis are ongoing for Ecospace, this figure shows potential maps (based on preliminary results) for food provisioning taking the biomass of hake as an example. Maps generated with ArcGIS 10.2.2 for desktop (http://www.esri.com/software/arcgis).

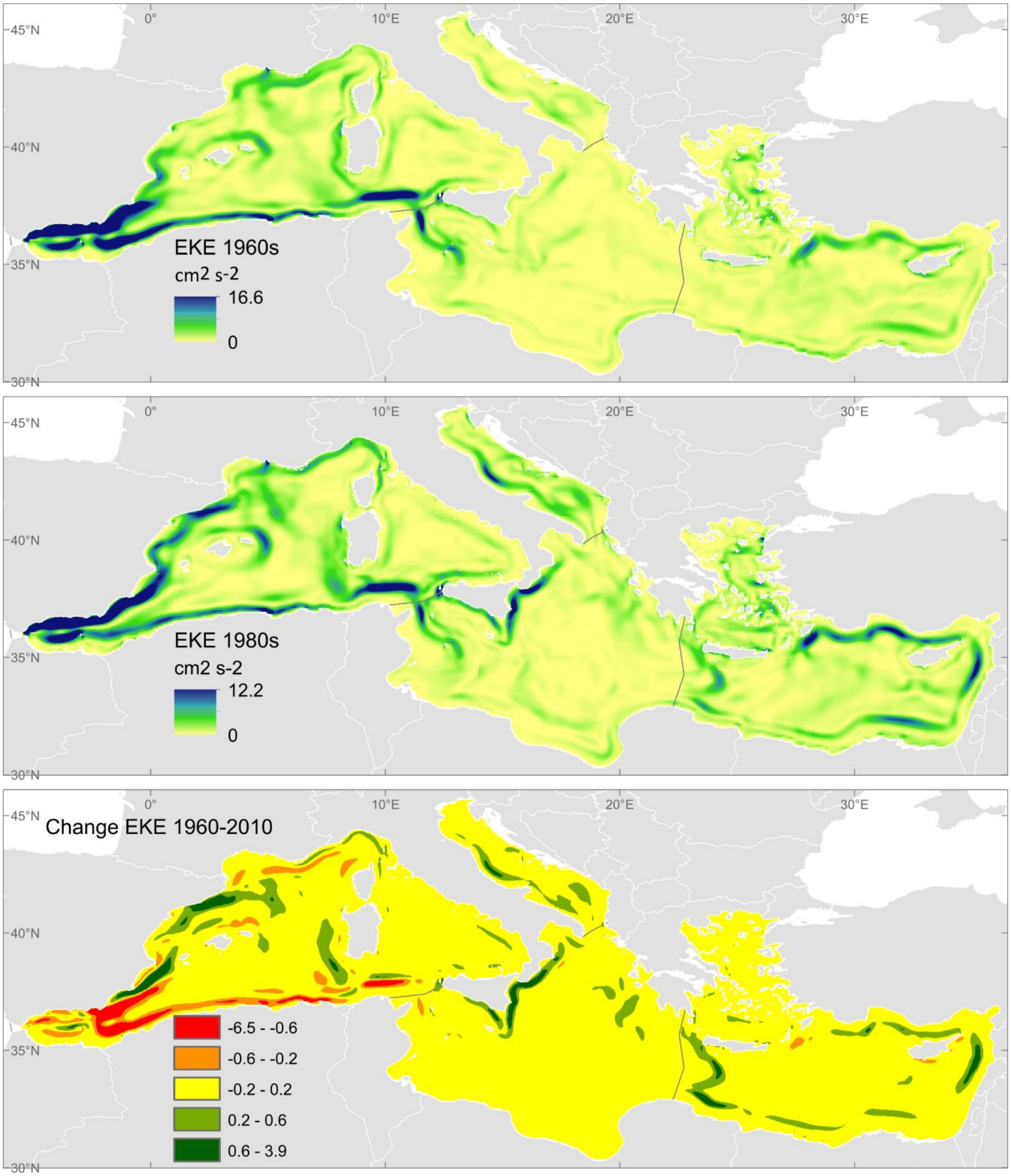

Supplementary Figure S3A. Distribution though time of the kinetic energy of surface currents (EKE), indicator of the capacity of water purification. Maps generated with ArcGIS 10.2.2 for desktop (http://www.esri.com/software/arcgis).

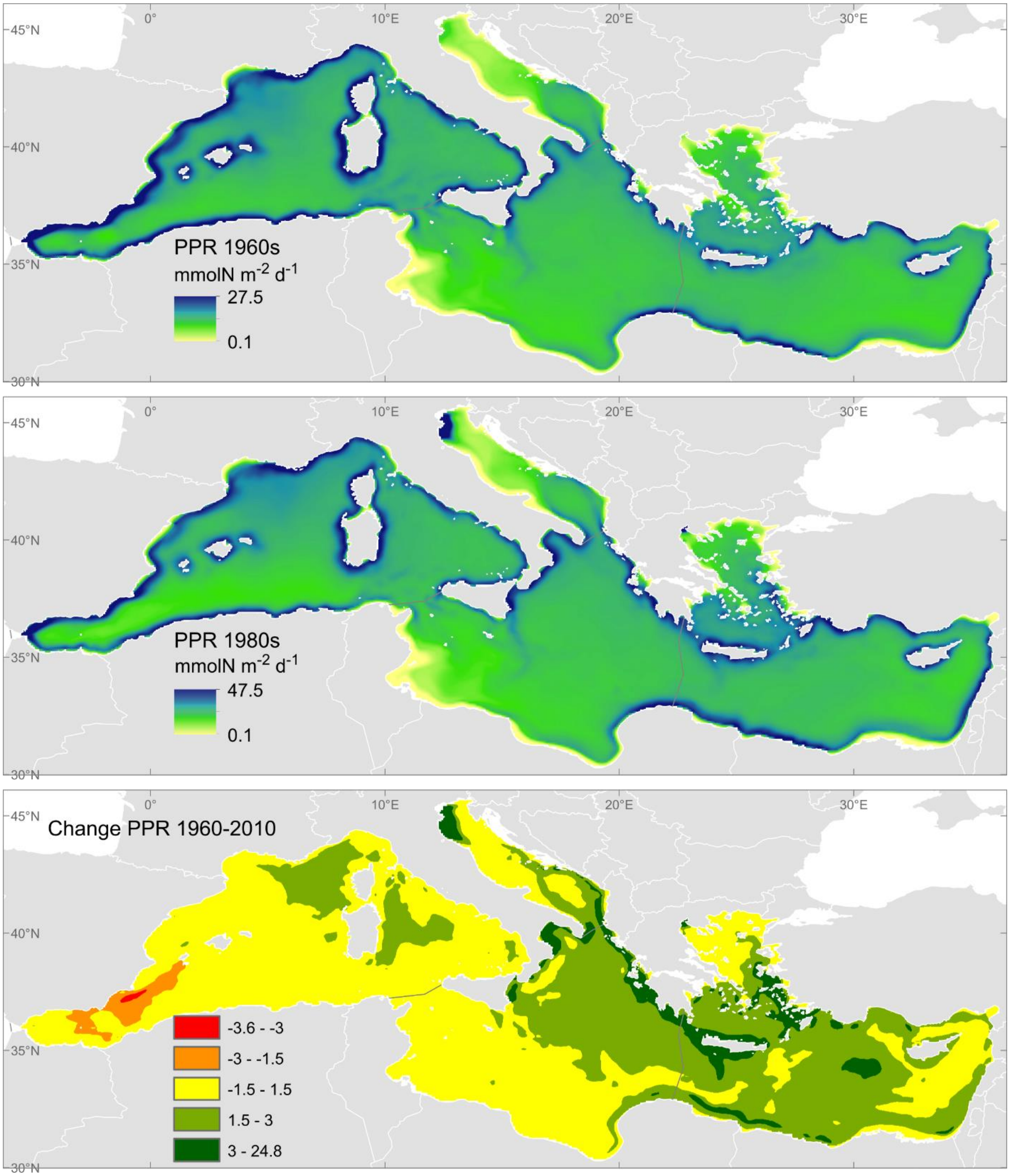

Supplementary Figure S3B. Distribution though time of primary production (PPR), indicator of the short-term flow of water purification. Maps generated with ArcGIS 10.2.2 for desktop (http://www.esri.com/software/arcgis*).*

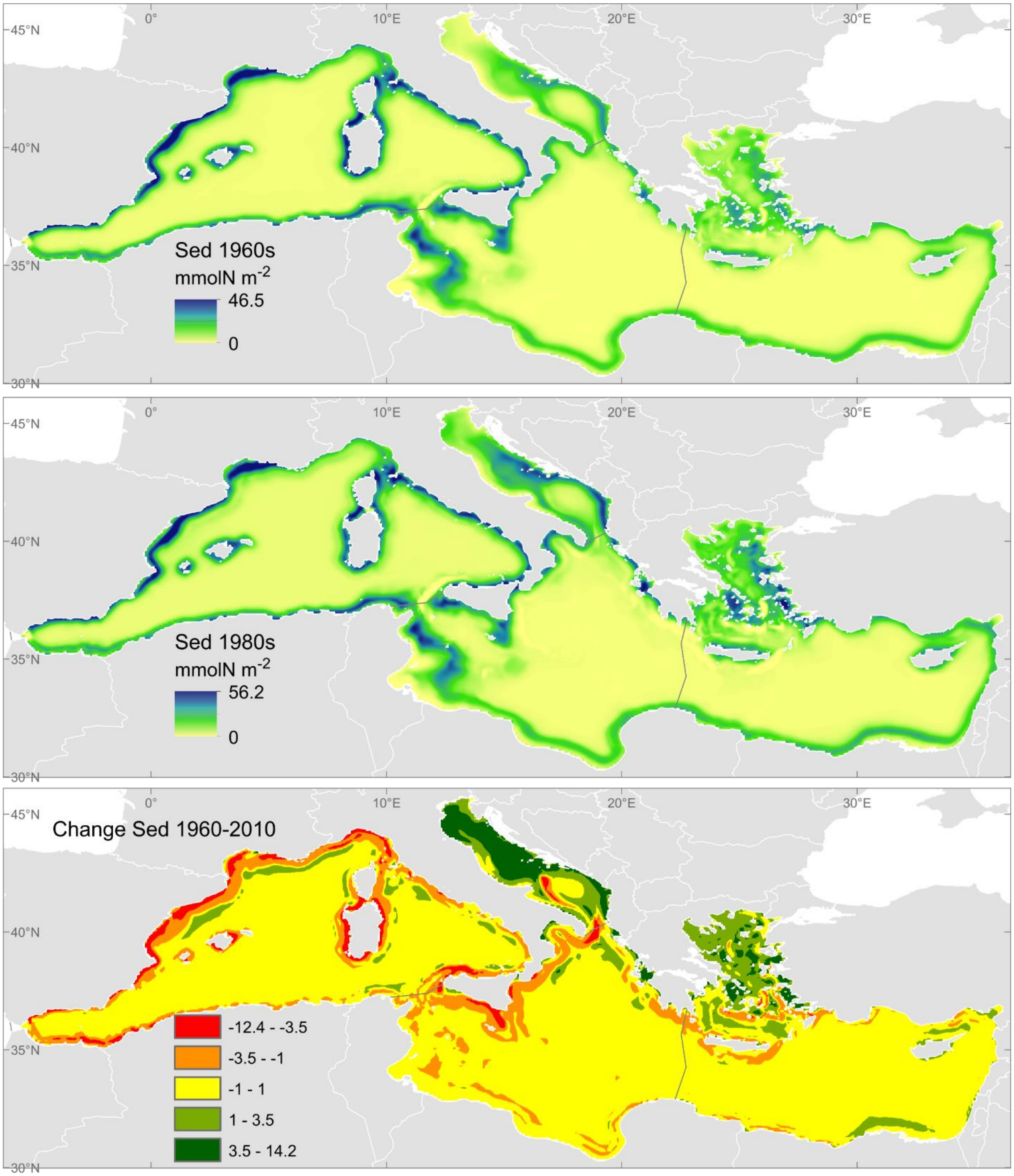

Supplementary Figure S3C. Distribution though time of the burial of organic matter into the sediments (Sed), indicator of the long-term flow of water purification. Maps generated with ArcGIS 10.2.2 for desktop (http://www.esri.com/software/arcgis).

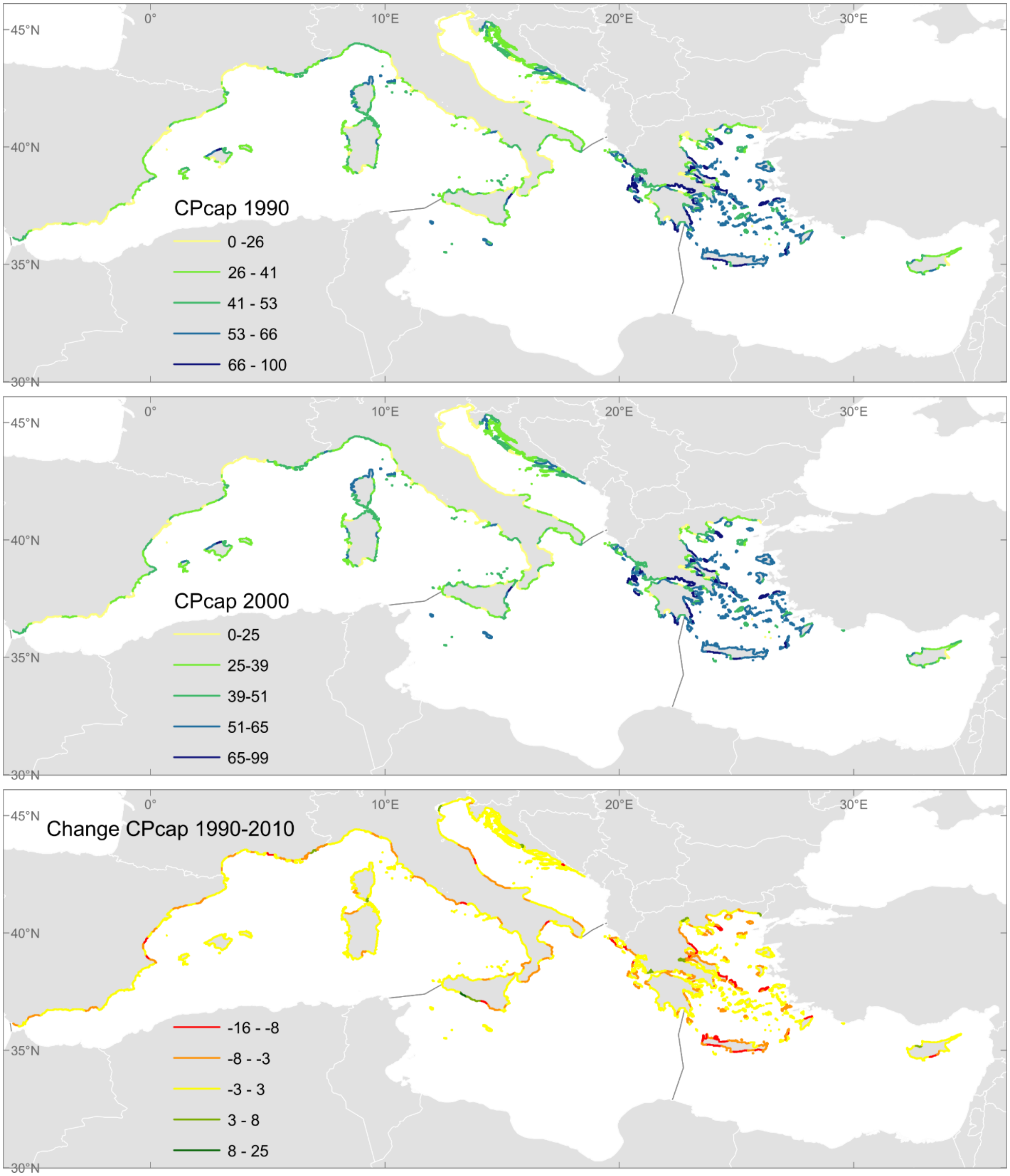

Supplementary Figure S4A. Distribution though time of the coastal protection capacity (CPcap), indicator of the natural capacity to provide flood and erosion protection. Maps generated with ArcGIS 10.2.2 for desktop (http://www.esri.com/software/arcgis*).*

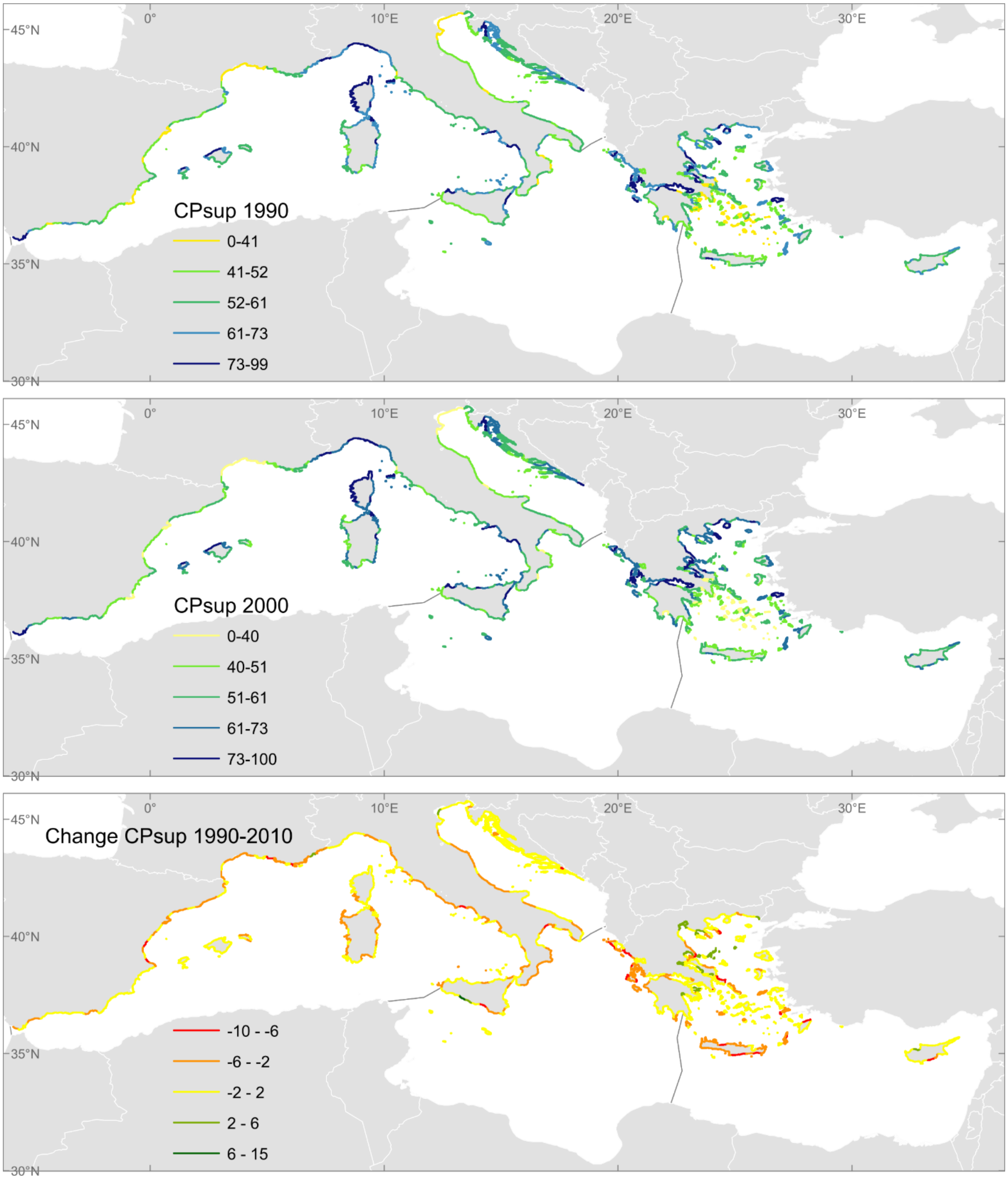

Supplementary Figure S4B. Distribution though time of the coastal protection supply (CPsup), indicator of the actual flow of coastal protection. Maps generated with ArcGIS 10.2.2 for desktop (http://www.esri.com/software/arcgis).

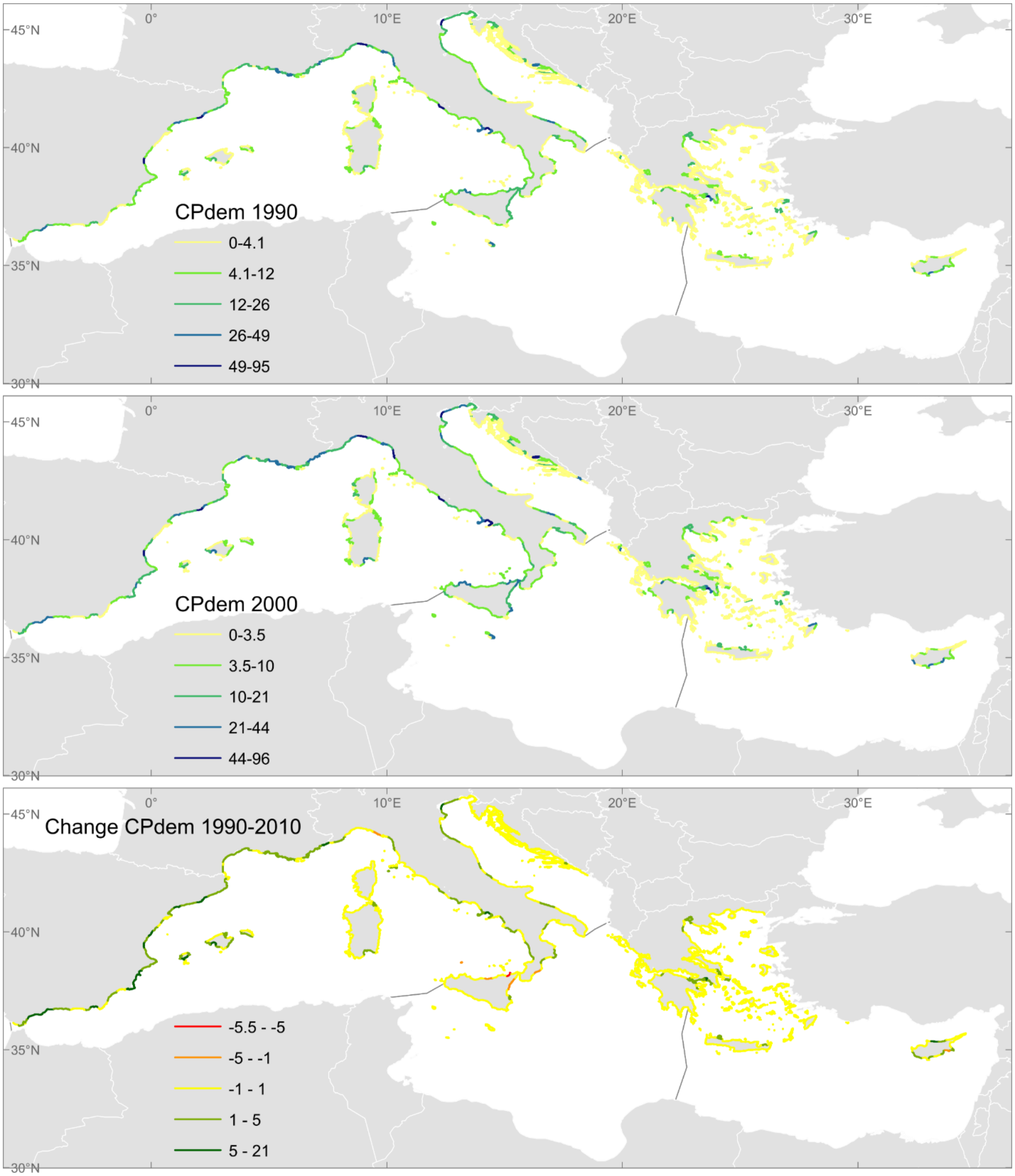

Supplementary Figure S4C. Distribution though time of the human demand for coastal protection (CPdem), indicator of the benefit from coastal protection. Maps generated with ArcGIS 10.2.2 for desktop (http://www.esri.com/software/arcgis).

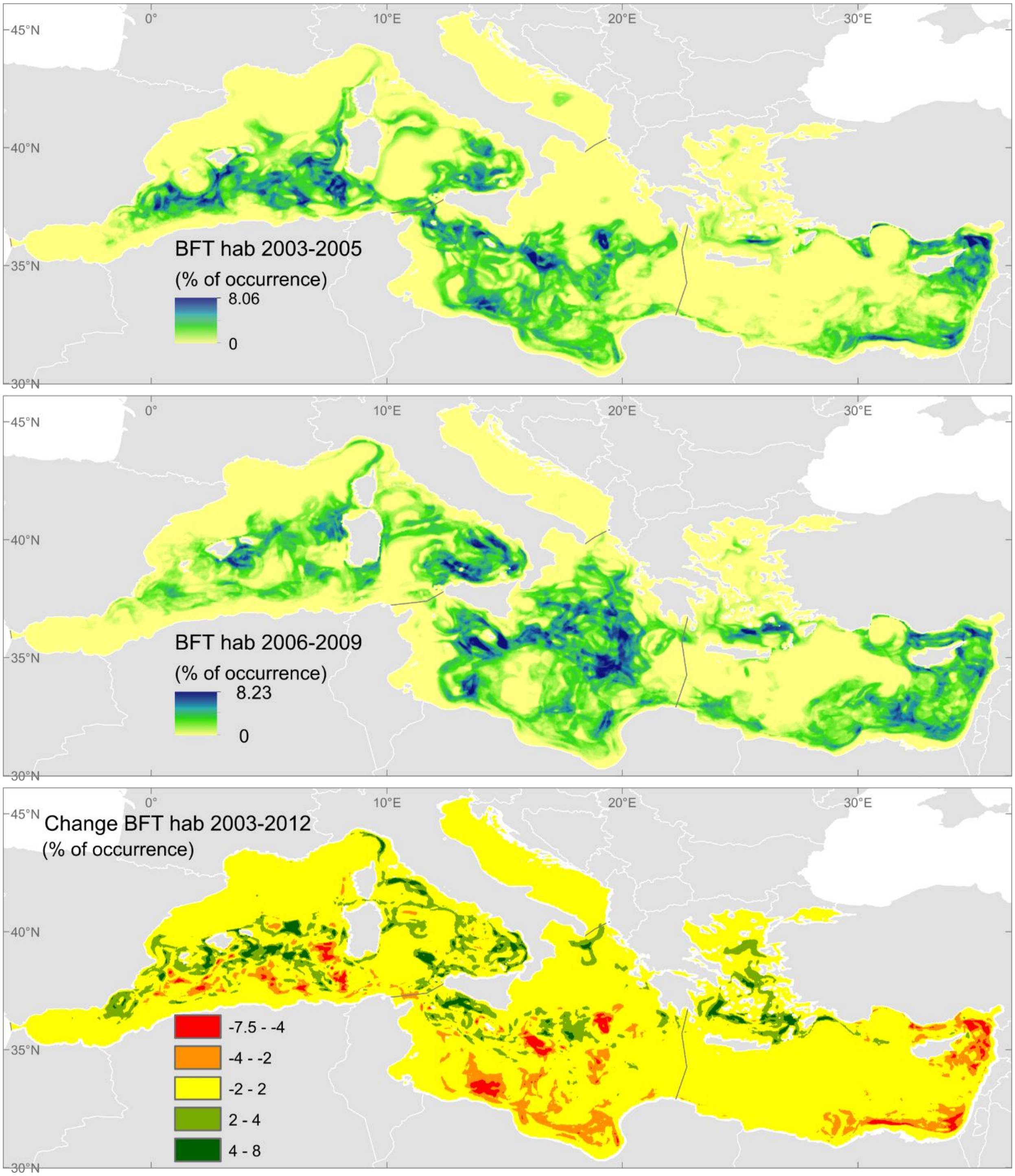

*Supplementary Figure S5A. Distribution though time of the favourable spawning habitat for bluefin tuna (BFT hab), indicator of the capacity of lifecycle maintenance. Maps generated with ArcGIS 10.2.2 for desktop (*http://www.esri.com/software/arcgis*).*

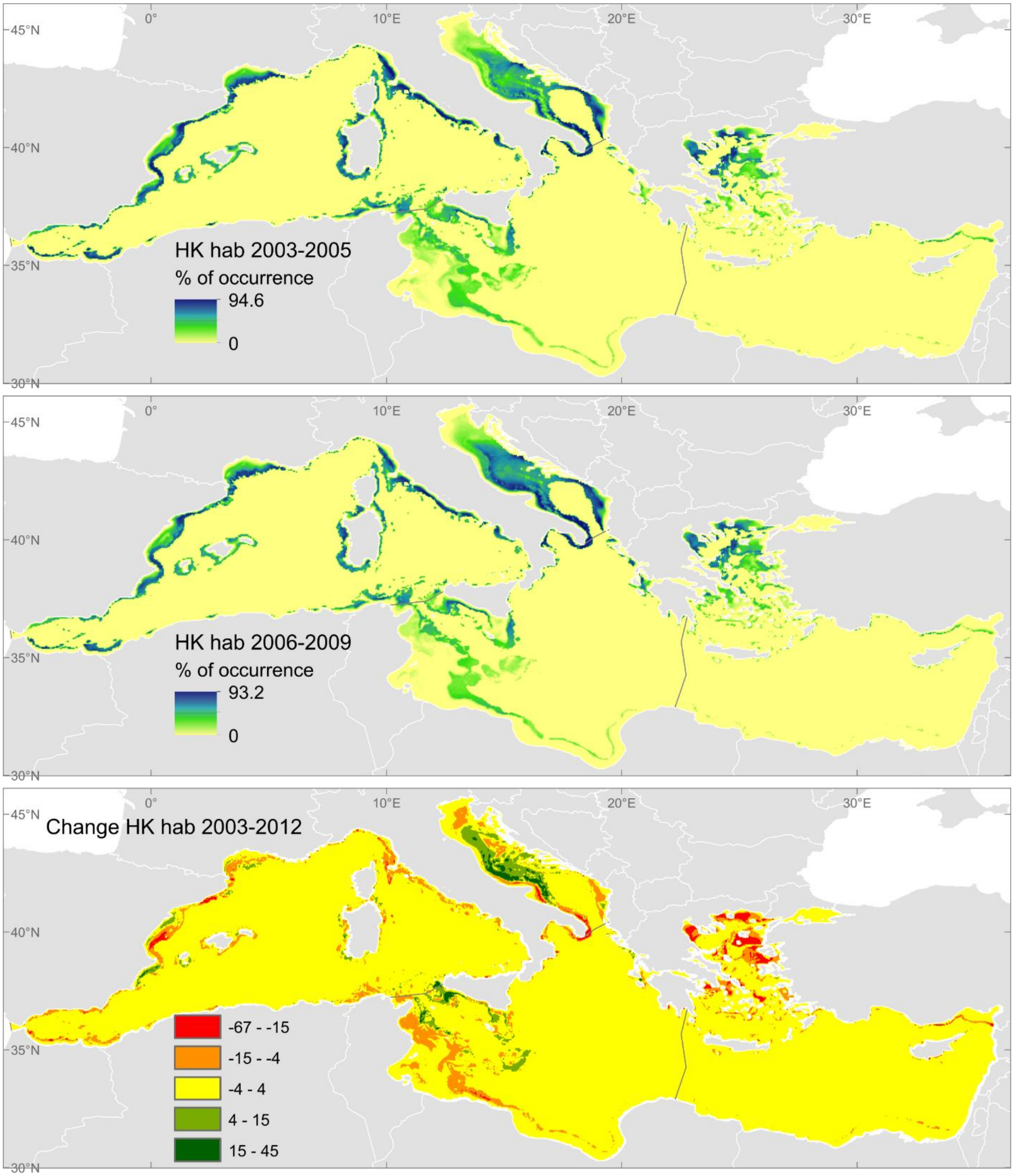

Supplementary Figure S5B. Distribution though time of the favourable nursery habitat for European hake (HK hab), indicator of the capacity of lifecycle maintenance. Maps generated with ArcGIS 10.2.2 for desktop (http://www.esri.com/software/arcgis).

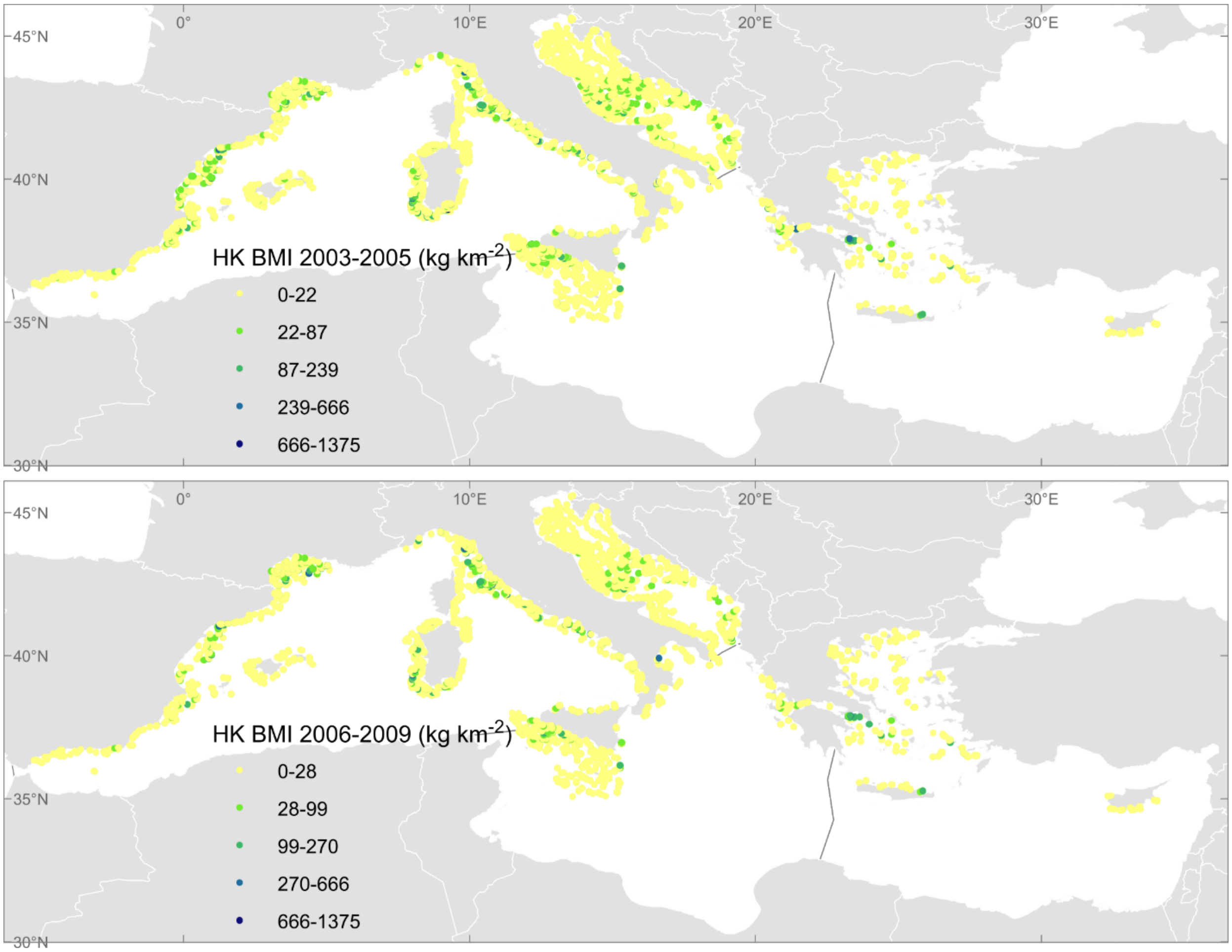

Supplementary Figure S5C. Distribution though time of the hake biomass index (HK BMI), indicator of the realized flow of lifecycle maintenance. Maps generated with ArcGIS 10.2.2 for desktop (http://www.esri.com/software/arcgis).

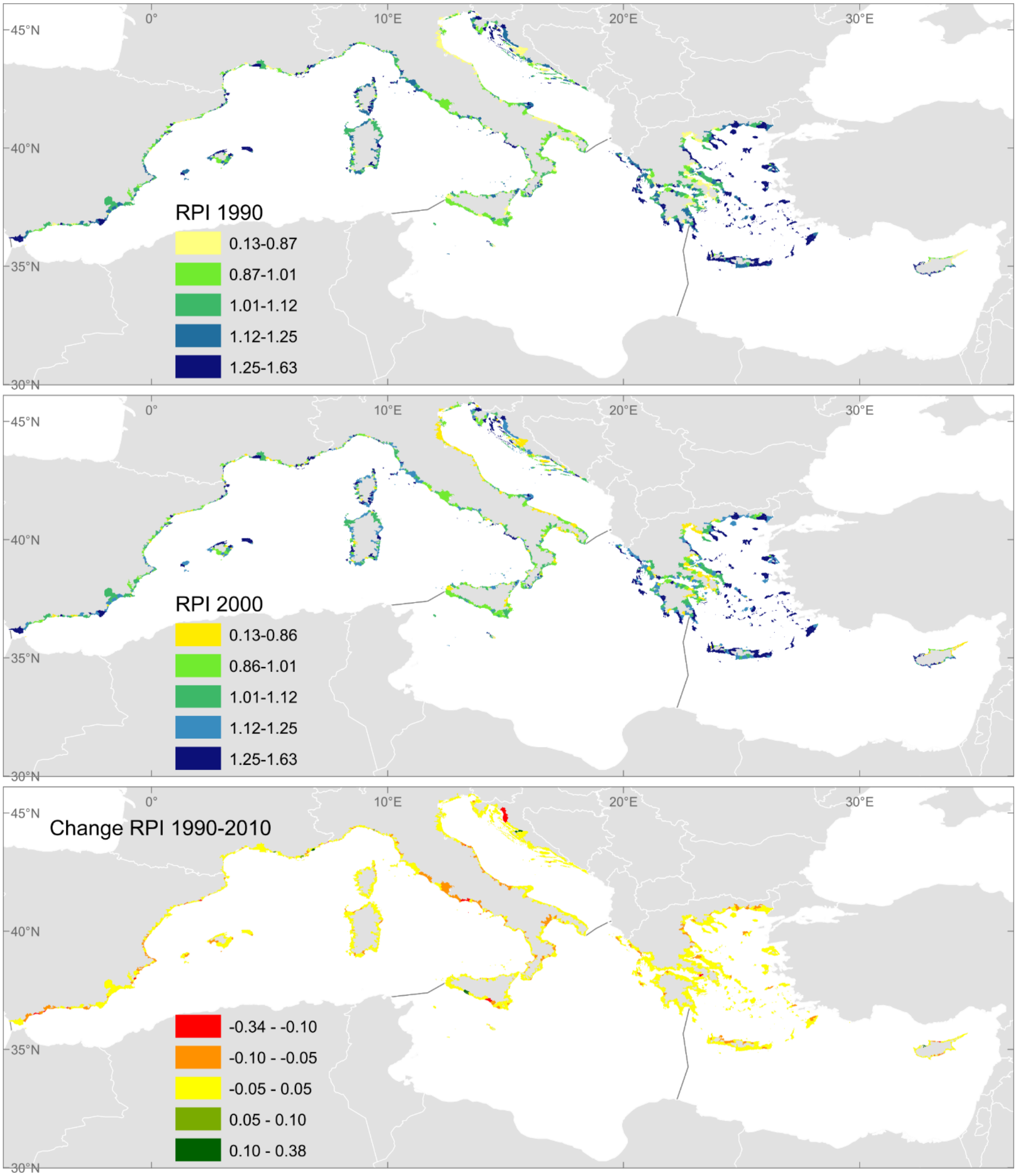

Supplementary Figure S6A. Distribution though time of the coastal recreation potential index (RPI), indicator of the capacity for nature-based recreation. Maps generated with ArcGIS 10.2.2 for desktop (http://www.esri.com/software/arcgis).

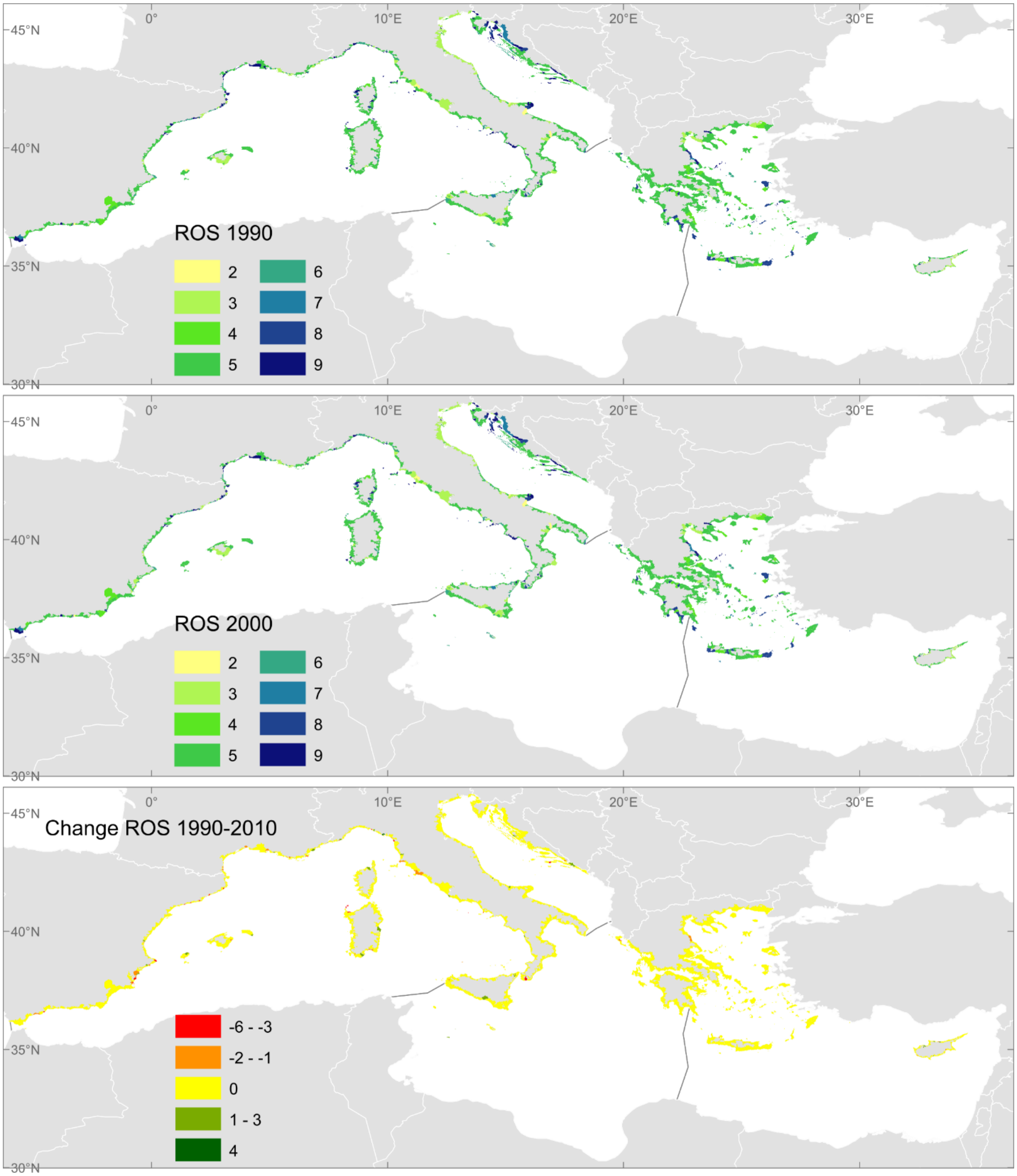

Supplementary Figure S6B. Distribution though time of the coastal recreation opportunity spectrum (ROS), indicator of the flow of recreation (2 is the minimum and 9 the maximum flow, see legend in Supplementary Table S5). Maps generated with ArcGIS 10.2.2 for desktop (http://www.esri.com/software/arcgis).

REFERENCES

1. de Groot, R. *et al.* in *The Economics of Ecosystems and Biodiversity: Ecological and Economic Foundations* (ed. Pushpam, K.) 9–40 (TEEB, 2010).

2. Haines-Young, R. & Potschin, M. in *Ecosystem Ecology: A New Synthesis* (eds. Raffaelli, D. & Frid, C.) 110–139 (BES Ecological Reviews Series, Cambridge University Press, 2010).

3. Christensen, V. & Walters, C. J. Ecopath with Ecosim: methods, capabilities and limitations. *Ecol. Modell.* **172,** 109–139 (2004).

4. Walters, C., Christensen, V. & Pauly, D. Structuring dynamic models of exploited ecosystems from trophic mass-balance assessments. *Rev. fish Biol. Fish.* **7,** 139–172 (1997).

5. Preikshot, D. B. The influence of geographical scale, climate and trophic dynamics upon North Pacific Oceanic ecosystem models. (University of British Columbia, Vancouver, 2007).

6. Christensen, V., Walters, C., Pauly, D. & Forrest, R. *Ecopath with Ecosim 6: A user's guide*. (2008).

7. Walters, C., Pauly, D. & Christensen, V. Ecospace: prediction of mesoscale spatial patterns in trophic relationships of exploited ecosystems, with emphasis on the impacts of marine protected areas. *Ecosystems* **2,** 539–554 (1999).

8. Christensen, V. *et al.* Representing variable habitat quality in a spatial food web model. *Ecosystems* **17,** 1397–1412 (2014).

9. Steenbeek, J. *et al.* Bridging the gap between ecosystem modeling tools and geographic information systems: Driving a food web model with external spatial–temporal data. *Ecol. Modell.* **263,** 139–151 (2013).

10. Kaschner, K., Watson, R., Trites, A. & Pauly, D. Mapping world-wide distributions of marine mammal species using a relative environmental suitability (RES) model. *Mar. Ecol. Prog. Ser.* **316,** 285–310 (2006).

11. Piroddi, C., Coll, M., Steenbeek, J., Macias Moy, D. & Christensen, V. Modelling the Mediterranean marine ecosystem as a whole: addressing the challenge of complexity. *Mar. Ecol. Prog. Ser.* **533,** 47–65 (2015).

12. Aguilar, A. *Current status of Mediterranean monk seal (Monachus monachus) populations*. (IUCN, 1998).

13. Sergeant, D., Ronald, K., Boulva, J. & Berkes, F. The recent status of Monachus monachus, the Mediterranean monk seal. *Biol. Conserv.* **14,** 259–287 (1978).

14. Panou, A., Jacobs, J. & Panos, D. The endangered Mediterranean monk seal Monachus monachus in the Ionian Sea, Greece. *Biol. Conserv.* **64,** 129–140 (1993).

15. Adamantopoulou, S. *et al.* Movements of Mediterranean monk seals (Monachus monachus) in the eastern Mediterranean Sea. *Aquat. Mamm.* **37,** 256 (2011).

16. Antolović, J. *et al.* Protection of the Mediterranean Monk Seal (Monachus monachus) and its habitats. in *Rapport du 36e Congrés de la Commission Internationale pour l'Exploration Scientifique de la mer*

Méditerranée. 36–230 (2001).

17. Notarbartolo di Sciara, G. *et al. National strategy and action plan for the conservation of the Mediterranean monk seal in Greece, 2009-2015*. *Publication prepared as part of the LIFE-Nature Project: MOFI: Monk Seal and Fisheries: Mitigating the conflict in Greek Seas* (2009).

18. Hellenic Society for the Study and Protection of the Monk sea. *Status of the Mediterranean monk seal Monachus monachus in Greece*. (2009).

19. Mo, G., Agnesi, S., Di Nora, T. & Tunesi, L. Mediterranean monk seal sightings in Italy through interviews: Validating the information (1998–2006). *Rapp. Comm. Int. Mer Méditerranée* **38,** 542 (2007).

20. Gomerčić, T., Huber, D., Gomerčić, M. Đ. & Gomerčić, H. Presence of the Mediterranean monk seal (Monachus monachus) in the Croatian part of the Adriatic Sea. *Aquat. Mamm.* **37,** 243–247 (2011).

21. Güçlüsoy, H., Kiraç, C. O., Veryeri, N. O. & Savas, Y. Status of the Mediterranean monk seal, Monachus monachus (Hermann, 1779) in the coastal waters of Turkey. *EU J. Fish. Aquat. Sci.* **21,** 201–210 (2004).

22. Gucu, A. C., Gucu, G. & Orek, H. Habitat use and preliminary demographic evaluation of the critically endangered Mediterranean monk seal (Monachus monachus) in the Cilician Basin (Eastern Mediterranean). *Biol. Conserv.* **116,** 417–431 (2004).

23. Scheinin, A. P., Goffman, O., Elasar, M., Perelberg, A. & Kerem, D. H. Mediterranean monk seal (Monachus monachus) resighted along the Israeli coastline after more than half a century. *Aquat. Mamm.* **37,** 241 (2011).

24. Casale, P. & Margaritoulis, D. *Sea turtles in the Mediterranean: distribution, threats and conservation priorities*. (IUCN, 2010).

25. Margaritoulis, D. & Rees, A. F. The loggerhead turtle, Caretta caretta, population nesting in Kyparissia Bay, Peloponnesus, Greece: results of beach surveys over seventeen seasons and determination of the core nesting habitat. *Zool. Middle East* **24,** 75–90 (2001).

26. Margaritoulis, D. *et al.* in *Loggerhead Sea Turtles* (eds. Bolten, A. & Witherington, B.) (Smithsonian Institution Press, 2003).

27. Mingozzi, T. *et al.* in *Biodiversity and Conservation in Europe* 277–299 (Springer, 2008).

28. Lauriano, G., Panigada, S., Casale, P., Pierantonio, N. & Donovan, G. Aerial survey abundance estimates of the loggerhead sea turtle Caretta caretta in the Pelagos Sanctuary, northwestern Mediterranean Sea. *Mar Ecol Prog Ser* **437,** 291–302 (2011).

29. Kasparek, M. & Baran, İ. *Marine turtles, Turkey: Status survey 1988 and recommendations for conservation and management*. (World Wide Fund for Nature, 1989).

30. Kasparek, M., Godley, B. J. & Broderick, A. C. Nesting of the green turtle, Chelonia mydas, in the Mediterranean: a review of status and conservation needs. *Zool. Middle East* **24,** 45–74 (2001).

31. Groombridge, B. *Marine turtles in the Mediterranean: distribution, population status, conservation*. *Nature and Environment* (Council of Europe, 1990).

32. Canbolat, A. F. A review of sea turtle nesting activity along the Mediterranean coast of Turkey. *Biol.*

Conserv. **116,** 81–91 (2004).

33. Camiñas, J. A. Sea turtles of the Mediterranean Sea: population dynamics, sources of mortality and relative importance of fisheries impacts. *FAO Fish. Rep.* 27–84 (2004).

34. Broderick, A. C. & Godley, B. J. Population and nesting ecology of the green turtle, Chelonia mydas, and the loggerhead turtle, Caretta caretta, in northern Cyprus. *Zool. Middle East* **13,** 27–46 (1996).

35. Bentivegna, F. *et al.* Loggerhead turtle (Caretta caretta) nests at high latitudes in Italy: a call for vigilance in the Western Mediterranean. *Chelonian Conserv. Biol.* **9,** 283–289 (2010).

36. Broderick, A., Glen, F., Godley, B. & Hays, G. Estimating the number of green and loggerhead turtles nesting annually in the Mediterranean. *Oryx* **36,** 227–235 (2002).

37. Ahrens, R. N. M., Walters, C. J. & Christensen, V. Foraging arena theory. *Fish Fish.* **13,** 41–59 (2012).

38. MEDIAS. *Report of 3rd meeting for MEDiterranean Acoustic Surveys (MEDIAS) in the framework of European Data Collection Framework*. (2010).

39. MEDIAS. *Report of 5th meeting for MEDIterranean Acoustic Surveys in the framework of European Data Collection Framework (DCF)*. (2012).

40. Sinovčić, G., Zorica, B., Keč, V. Č. & Mustać, B. Inter-annual fluctuations of the population structure, condition, length-weight relationship and abundance of sardine, Sardina pilchardus (Walb., 1792), in the nursery and spawning ground (coastal and open sea waters) of the eastern Adriatic Sea (Croatia). *Acta Adriat.* **50,** 11–21 (2009).

41. Santojanni, A. *et al.* Stock assessment of sardine (Sardina pilchardus, Walb.) in the Adriatic Sea with an estimate of discards. *Sci. Mar.* **69,** 603–617 (2005).

42. Bedairia, A. & Djebar, A. B. A preliminary analysis of the state of exploitation of the sardine, Sardina pilchardus (Walbaum, 1792), in the gulf of Annaba, East Algerian. *Anim. Biodivers. Conserv.* **32,** 89–99 (2009).

43. Patti, B. *et al.* Interannual fluctuations in acoustic biomass estimates and in landings of small pelagic fish populations in relation to hydrology in the Strait of Sicily. *Chem. Ecol.* **20,** 365–375 (2004).

44. García, A. & Giraldez, A. *Small pelagic fish research in the Mediterranean by the Spanish Institute of Oceanography: available data series for a climatic analysis*. *Report of the Working Group on Small Pelagic Fishes, their Ecosystems and Climate Impact (WGSPEC)* (2012).

45. De Felice, A. *Biomass evaluation of anchovy (E. encrasicolus), sardine (S. pilchardus) and sprat (S. sprattus) in the western Adriatic Sea by means of acoustics and preliminary analysis of possible relationships with environmental parameters*. *Report of the Working Group on Small Pelagic Fishes, their Ecosystems and Climate Impact (WGSPEC)* (2012).

46. STECF. *Assessment of Mediterranean Sea stocks part I*. *EUR 24637 EN, JRC 62020* (Publications Office of the European Union, 2010).

47. STECF. *Assessment of Mediterranean Sea stocks part I (STECF 11-08)*. *EUR 24902 EN, JRC 66052* (Publications Office of the European Union, 2011).

48. STECF. *Assessment of Mediterranean Sea stocks part I (STECF 12-19)*. *EUR 25602 EN, JRC 76735*

(Publications Office of the European Union, 2012).

49. STECF. *Assessment of Mediterranean Sea stocks part I (STECF 13-22). EUR 26329 EN, JRC 86087* (Publications Office of the European Union, 2013).

50. STECF. *Assessment of Mediterranean Sea stocks part II. EUR 24810 EN, JRC 64927* (Publications Office of the European Union, 2010).

51. STECF. *Assessment of Mediterranean Sea stocks part II (STECF 11-14). EUR 25053 EN, JRC 67797* (Publications Office of the European Union, 2011).

52. STECF. *Assessment of Mediterranean Sea stocks part II (STECF 13-05). EUR 25309 EN, JRC 81592* (Publications Office of the European Union, 2012).

53. STECF. *Assessment of Mediterranean Sea stocks part II (STECF-14-08). EUR 26614 EN, JRC 89860* (Publications Office of the European Union, 2013).

54. Wei, C.-L. *et al.* Global patterns and predictions of seafloor biomass using random forests. *PLoS One* **5,** e15323 (2010).

55. Liquete, C., Zulian, G., Delgado, I., Stips, A. & Maes, J. Assessment of coastal protection as an ecosystem service in Europe. *Ecol. Indic.* (2013).

56. EUROSION. The Coastal Erosion Layer. Geomorphology, geology, erosion trends and coastal defence works, Version 2. (2004). Available at: http://www.eea.europa.eu/data-and-maps; http://www.eurosion.org/.

57. Tempera, F. *Bringing together harmonized EUNIS seabed habitat geospatial information for the European Seas. JRC Scientific and Technical Research series, EUR 27237 EN*. (Publications Office of the European Union, 2015).

58. Eurostat. GEOSTAT population grid 2011. (2014). Available at: http://ec.europa.eu/eurostat/web/gisco/geodata/reference-data/population-distribution-demography.

59. Eurostat. Population change - Demographic balance and crude rates at regional level (NUTS 3). Population database. (2012). Available at: http://ec.europa.eu/eurostat/web/products-datasets/-/demo_r_d3dens.

60. Vafeidis, A. T. *et al.* A New Global Coastal Database for Impact and Vulnerability Analysis to Sea-Level Rise. *J. Coast. Res.* **24,** 917–924 (2008).

61. Pujol, M.-I. *et al.* A 20-year reference period for SSALTO/DUACS products. in *Ocean Surface Topography Science Team meeting* (2013).

62. Carrère, L., Lyard, F., Cancet, M., Guillot, A. & Roblou, L. FES2012: A new global tidal model taking advantage of nearly twenty years of altimetry. in *Proceedings of the symposium '20 years of Progress in Radar Altimetry'* (2012).

63. Druon, J.-N. *et al.* Modelling of European hake nurseries in the Mediterranean Sea: An ecological niche approach. *Prog. Oceanogr.* **130,** 188–204 (2015).

64. Druon, J.-N. *et al.* Habitat suitability of the Atlantic bluefin tuna by size class: An ecological niche

approach. *Prog. Oceanogr.* **142,** 30–46 (2016).

65. *Mediterranean marine demersal resources: the Medits international trawl survey (1994-1999)*. (eds Abelló, P. *et al.*) (Sci. Mar. 66 (Suppl. 2), 2002).

66. Paracchini, M. L. *et al.* Mapping cultural ecosystem services: A framework to assess the potential for outdoor recreation across the EU. *Ecol. Indic.* **45,** 371–385 (2014).

67. Zulian, G., Paracchini, M. L., Maes, J. & Liquete, C. *ESTIMAP: Ecosystem services mapping at European scale*. (2013).

68. Eagles, P., McCool, S. & Haynes, C. *Sustainable tourism in protected areas: Guidelines for planning and management*. (World Commission on Protected Areas (WCPA), Best Practice Protected Area Guidelines series 8, 2002).

69. Roberts, J. J., Best, B. D., Dunn, D. C., Treml, E. A. & Halpin, P. N. Marine Geospatial Ecology Tools: An integrated framework for ecological geoprocessing with ArcGIS, Python, R, MATLAB, and C++. *Environ. Model. Softw.* **25,** 1197–1207 (2010).
